# The global serological prevalence of *Toxoplasma gondii* in felids during the last five decades (1967–2017): a systematic review and meta-analysis

**DOI:** 10.1186/s13071-020-3954-1

**Published:** 2020-02-17

**Authors:** Mahbobeh Montazeri, Tahereh Mikaeili Galeh, Mahmood Moosazadeh, Shahabeddin Sarvi, Samira Dodangeh, Javad Javidnia, Mehdi Sharif, Ahmad Daryani

**Affiliations:** 1 Toxoplasmosis Research Center, Mazandaran University of Medical Sciences, Sari, Iran; 2 Department of Parasitology, Sari Medical School, Mazandaran University of Medical Sciences, Sari, Iran; 3 Student Research Committee, Mazandaran University of Medical Sciences, Sari, Iran; 4 Health Sciences Research Center, Addiction Institute, Mazandaran University of Medical Sciences, Sari, Iran; 5 Department of Medical Mycology, School of Medicine, Mazandaran University of Medical Sciences, Sari, Iran

**Keywords:** *Toxoplasma gondii*, Serology, Domestic cat, Wild cat, Meta-analysis

## Abstract

**Background:**

Felids (domestic and wild cats) are important in the epidemiology of the parasite *Toxoplasma gondii* because they are the only hosts that can excrete the environmentally resistant oocysts. We conducted a systematic review and meta-analysis to estimate the global prevalence of *T. gondii* in species of the family Felidae.

**Methods:**

We searched seven databases (PubMed, Embase, Google Scholar, ScienceDirect, Scopus, Proquest and Web of Science) for studies reporting seroprevalence of *T. gondii* in felids from 1967 to 31 December 2017. A total of 217 published papers, containing 223 datasets were eligible for inclusion in the meta-analysis, comprised 59,517 domestic and 2733 wild cats from 1967 to 2017.

**Results:**

The pooled global *T. gondii* seroprevalence was estimated to be 35% (95% CI: 32–38%) and 59% (95% CI: 56–63%) in domestic cats and wild felids, respectively, using random effects model. The seroprevalence was higher in Australia and Africa where the *T. gondii* seropositivity in domestic cats was 52% (95% CI: 15–89%) and 51% (95% CI: 20–81%), respectively. The lowest seroprevalence was estimated in Asia 27% (95% CI: 24–30%). The seroprevalence values for *T. gondii* in wild felids were 74% (95% CI: 62–83%) in Africa, 67% (95% CI: 23–111%) in Asia, 67% (95% CI: 58–75%) in Europe and 66% (95% CI: 41–91%) in South America.

**Conclusions:**

Our study provides the global prevalence of *T. gondii* in species of the family Felidae and is a source of information to aid public health workers in developing prevention plans.
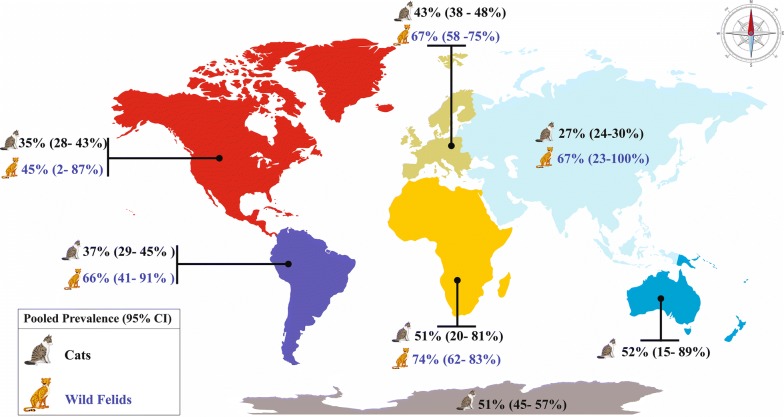

## Background

*Toxoplasma gondii* is a ubiquitous apicomplexan parasite responsible for a neglected parasitic disease, toxoplasmosis, in a wide variety of animals such as birds, livestock, and the great majority of homoeothermic vertebrates, including humans, worldwide acting as intermediate hosts [1, 2]. Based on formal reports, over one billion people in the world are estimated to be infected with *T. gondii* [3], which is transmitted mainly by ingestion of food, water, vegetables and fruits contaminated with sporulated oocysts shed from cats or ingesting tissue cysts from raw or undercooked meat [1]. *Toxoplasma gondii* prevalence in humans is different among different countries and in some regions can be high (e.g. Brazil, 77.5%; Sao Tome and Principe, 75.2%; Iran, 63.9%; Colombia, 63.5%; and Cuba, 61.8%) [4].

The Centers for Disease Control and Prevention (CDC) reported that toxoplasmosis is the second most common cause of death due to food-borne diseases (an estimated 327 deaths) and the fourth leading cause of hospitalizations attributable to food-borne illness (an estimated 4428 hospitalizations) from the mid to late 2000s in the USA [5].

The only definitive hosts of *T. gondii*, the members of the family Felidae (i.e. domestic cats and other felids), are the key animal species for the maintenance of this parasite [6]. This ubiquitous parasitic protozoan has three infectious forms: sporozoites (in oocysts), tachyzoites (rapidly multiplying form) and bradyzoites (tissue cyst form) [1]. The felids usually acquire *T. gondii* infection by orally ingesting meat containing viable *T. gondii* tissue cysts [7]. After ingestion, bradyzoites released from tissue cysts penetrate the epithelial cells of the intestinal tissues and initiate the formation of numerous asexual generations before the sexual cycle begins. *Toxoplasma gondii* completes its sexual life-cycle in the intestine of infected cats and millions of oocysts may be excreted into the environment between 3 and 18 days after infection, for several months resulting in the spread of toxoplasmosis to humans and animals [8, 9].

*Toxoplasma gondii* infection is important both in the veterinary and human medicine. It causes significant economic losses in terms of abortion in sheep and goats [10, 11]. Infections in healthy humans are usually asymptomatic; however, it is considered to be an opportunistic and life-threatening parasite in immunocompromised individuals and newborns [8].

Considering the public health and economic importance of toxoplasmosis, in this study, we provide the first systematic review and meta-analysis to evaluate the global seroprevalence of *T. gondii* in the family Felidae.

## Methods

The study was conducted according to the Preferred Reporting Items for Systematic Reviews and Meta-Analyses Protocols (PRISMA) for meta-analyses and systematic reviews of observational studies as described previously (Additional file 1: Table S1) [12].

### Search strategy

In this systematic review and meta-analysis, we searched databases (PubMed, Embase, Google Scholar, ScienceDirect, Scopus, Proquest and Web of Science) for studies reporting seroprevalence of *T. gondii* in felines from 1967 to 31 December 2017. The searches were restricted to articles in English. The main MeSH terms used in electronic searches were: (Cat OR *Felis*) AND (*Toxoplasma gondii* OR toxoplasmosis) AND (Sero OR seroprevalence OR serology).

The citations were imported into EndNote X7.4 for management. After removing duplicate records; two reviewers (MM and SD) independently screened the title and abstract of each article and made the final article selection. A third reviewer (TM) was consulted in case of uncertainty or disagreement between the two reviewers. Reference lists of retrieved citations and published reviews were also searched for additional studies.

### Inclusion and exclusion criteria

The cross-sectional studies were included if they investigated seroprevalence of *T. gondii* in felids providing original data and/or presented data that allowed us to assess the prevalence of *T. gondii* infection based on serological methods using serum identified up to 2017.

We excluded reviews, repeated studies, or human studies, as well as studies of animals which had been experimentally infected; non-serological investigations; studies with unclear testing methods, sample sizes of less than 25 felids [13], and lack of access to full article or insufficient data in the abstract.

### Data extraction and study quality assessment

From each selected study, the following information was collected: continent, country, year of publication, the first author, the serological test used, the reported cut-off, period of sampling, sampling season, number of felids, number of seropositive felids, percent of positive felids (according the IgG results for male, female and total felids, respectively), type of felid and quality score of each study. The extracted data were collated into an Excel table and compared, any disagreements in the results were resolved by discussion, and the final data were checked by three of the authors (TM, MM and SD).

A quality assessment of included studies was performed as described previously [14]. The following items were examined and given a score based on a simple scale system (2 for “yes”, 0 for “no”, or 1 for “unsure”): (i) Was the research objective clearly described?; (ii) Was the sampling method defined in detail?; (iii) Was the period of study clearly stated? (iv) Was the serological test method clearly pointed out?; (v) Were the subjects categorized into different subgroups?

### Meta-analysis

The seroprevalence of *T. gondii* and 95% confidence intervals (CI) were estimated for each study by using STATA version 11 (STATA Corp., College Station, Texas). In all statistical analyses, the significance level was considered at *P* < 0.05.

In this study, we estimated the seroprevalence of *T. gondii* for continents by synthesizing the seroprevalence rates of all studies from each continent. The ratio of positive samples to total samples was defined as seroprevalence. The forest plot was used to presenting results of the meta-analysis in wild felids [15].

*I*^2^ statistic was applied to assess the heterogeneity and inconsistency in the studiesʼ results [16]. *I*^2^ ranges between 0 and 100%, and values of ≥ 50% were considered as indicators of high heterogeneity and inconsistency. Given that *I*^2^ was substantial in this study; therefore, we used a random effects model at a 95% CI, to give a more conservative estimate of the global *T. gondii* seroprevalence. Also, publication bias was employed amongst the selected studies, by applying the Egger’s publication bias method [17]. Furthermore, to determine the source of heterogeneity, subgroup analyses were performed. In a subgroup analysis, we assessed the seroprevalence of *T. gondii* in males and females, and different geographical locations, South America, North America, Asia, Africa, Europe, Australia and Antarctica.

## Results

### Study characteristics

**Fig. 1:**
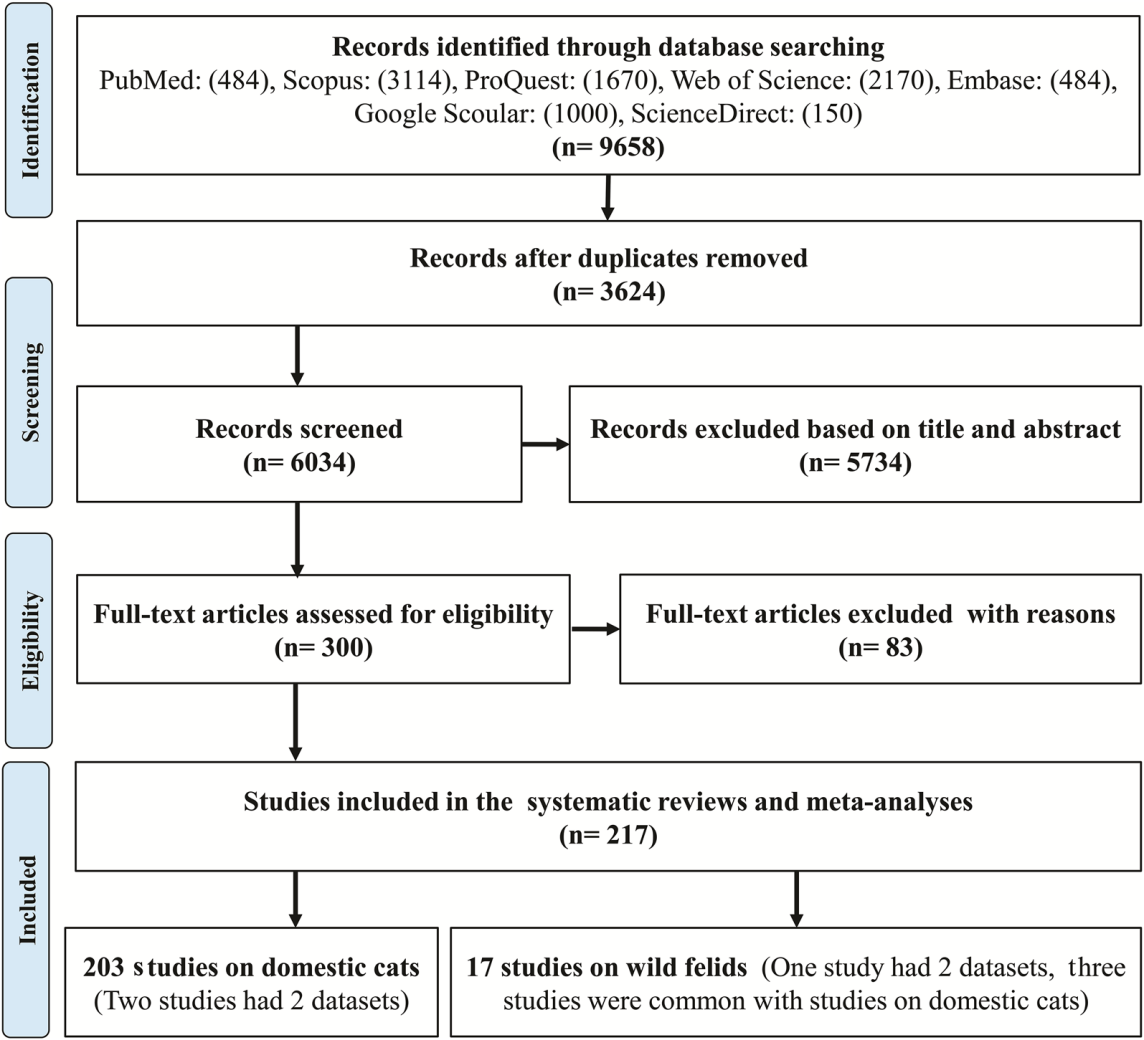
PRISMA chart of the study design process

Of 9658 studies from the literature review from seven databases, 217 studies [203 studies on domestic cats (two studies containing 2 datasets) and 17 studies on wild felids (one study containing 2 datasets, three studies were common with studies in domestic cats)] had eligibility to be accounted in the systematic review and meta-analysis according to the inclusion criteria (Fig. 1).

A total of 59,517 domestic cats and 2733 wild felids were evaluated for *T. gondii* antibodies from 1967 to 31 December 2017 in different geographical locations worldwide. Our final sample for domestic cats included seven continents, Antarctica (1 study), Africa (10 studies), Asia (79 studies, containing 81 datasets), Australia (3 studies), Europe (45 studies), North America (31 studies) and South America (34 studies). The final sample for wild felids included five continents, Africa and Europe (1 study), Asia (3 studies containing 4 datasets), North America (6 studies) and South America (6 studies containing 5 datasets).

**Fig. 2:**
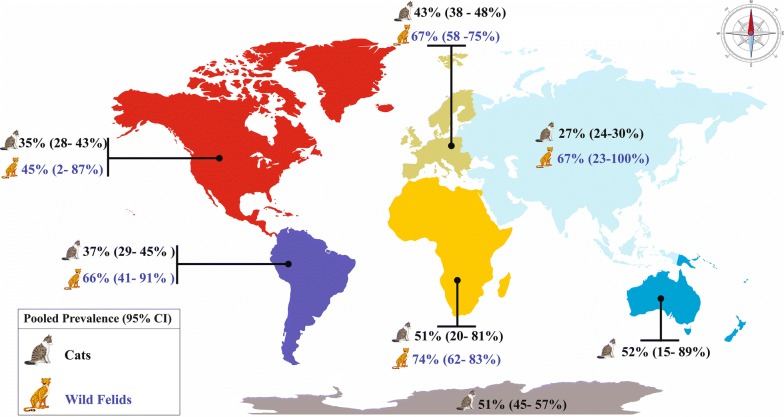
Worldwide *Toxoplasma gondii* seroprevalence in domestic cats and wild felids from different continents. Data are reported as mean (range)

A map summarizing the seroprevalence of *T. gondii* in domestic cats and wild felids in different continents is shown in Fig. 2. The countries with the highest number of reports in domestic cats were Brazil (29 studies), China (17 studies), the USA (15 studies), Japan (13 studies) and Iran (11 studies) (Additional file 2: Table S2). Also, Brazil (5 studies) had the highest number of reports in wild felids (Table 1). Of the 217 studies, 99 and 5 studies included data for the sex of the domestic cats (11,809 male and 13,413 female) in 6 continents and of the wild felids (798 male and 680 female) in 2 continents.

**Table 1:** Main features of studies regarding the global seroprevalence of *Toxoplasma gondii* in wild felids

Country	Publication year	Method	Cut-off	Period of sampling	Sample size	Prevalence (%)	Wild felid species	Quality score	References
Southern Africa	1999	IFAT	≥ 1:50	1984–1996	68	73.50	Non-domestic captive and free-ranging felids	8	Cheadle et al. [25]
Thailand	2006	LAT	≥ 1:64	2002–2004	136	15.40	Captive felids	8	Thiangtum et al. [26]
UAE	2008	MAT	≥ 1:25	2001–2008	36	86.11	Gordon’s wild, captive-born and wild-caught felids	8	Pas & Dubey [27]
Qatar	2010	MAT	≥ 1:25	2009	27	77.78	Arabian sand cat, African wild cat, cheetah, king cheetah	8	Dubey et al. [28]
UAE	2010	MAT	≥ 1:25	2009	53	90.60	Gordon’s wild cat, Arabian leopard, cheetah, caracal, African caracal	8	Dubey et al. [28]
France	2013	MAT	≥ 1:48	1996–2006	112	67.00	Wild cats	10	Afonso et al. [29]
USA (Alaska)	2001	MAT	≥ 1:25		255	15.30	Lynx	8	Zarnke et al. [30]
Canada	2001	MAT	≥ 1:25	1997–1998	116	44.00	Lynx, bobcats	10	Labelle et al. [31]
USA	2003	IFAT	≥ 1:50		101	44.55	Captive and free-ranging non-domestic felids	6	Spencer et al. [32]
Midwestern USA	2008	MAT	≥ 1:25	2003–2005	107	38.30	Cheetah, lynx, clouded leopard, African lion, jaguar, Amur leopard, Persian leopard, Amur tiger, fishing and Pallasʼs cats, puma, Texas puma, snow leopard	10	De Camps et al. [33]
USA (Mississippi)	2017	MAT	≥ 1:25	2014	35	100	Bobcats	10	Verma et al. [34]
America	2004	LAT	≥ 1:64	1984–1999	496	25.80	Pumas and bobcats	10	Kikuchi et al. [35]
Brazil	2001	MAT	≥ 1:20	1996–2000	37	64.90	European lynx, jungle cat, serval	10	Silva et al. [36]
Brazil	2001	MAT	≥ 1:20	1995–2001	865	54.60	Captive Neotropical felids	10	Silva et al. [37]
Brazil	2010	IFAT	≥ 1:40		161	63.40	Wild felids in Brazilian zoos	6	Andre et al. [38]
Brazil	2010	MAT	≥ 1:16		57	66.67	Neotropical felids (leopardus and puma)	8	Ullmann et al. [39]
Argentina	2012	ELISA	≥ 1:48	2000–2008	40	47.50	Geoffroy’s cats	9	Uhart et al. [40]
Brazil	2015	MAT	≥ 1:25	2000–2009	31	100	Free-ranging jaguars	8	Furtado et al. [41]

*Abbreviations:* IFAT, indirect fluorescent antibody test; LAT, latex agglutination test; MAT, modified agglutination test; ELISA, enzyme-linked immunosorbent assay

The enzyme-linked immunosorbent assay (ELISA), the modified agglutination test (MAT) and the immunofluorescence assay test (IFAT) were the serological methods used in 56, 53 and 41 studies on domestic cats and in 1, 11 and 3 studies on wild felids, respectively. Given that very different serological methods were used in the studies (Table 1, Additional file 2: Table S2), we did not carry out subgroup analysis based on diagnostic methods.

The results of the literature search and characteristics of each study are provided in Table 1 and Additional file 2: Table S2. The list of countries with no data on *T. gondii* infection in domestic cats and wild felids according to inclusion criteria in this study is provided in Additional file 3: Table S3 and Additional file 4: Table S4, respectively.

### Meta-analysis results

#### Pooled global seroprevalence of T. gondii in felids

**Fig. 3:**
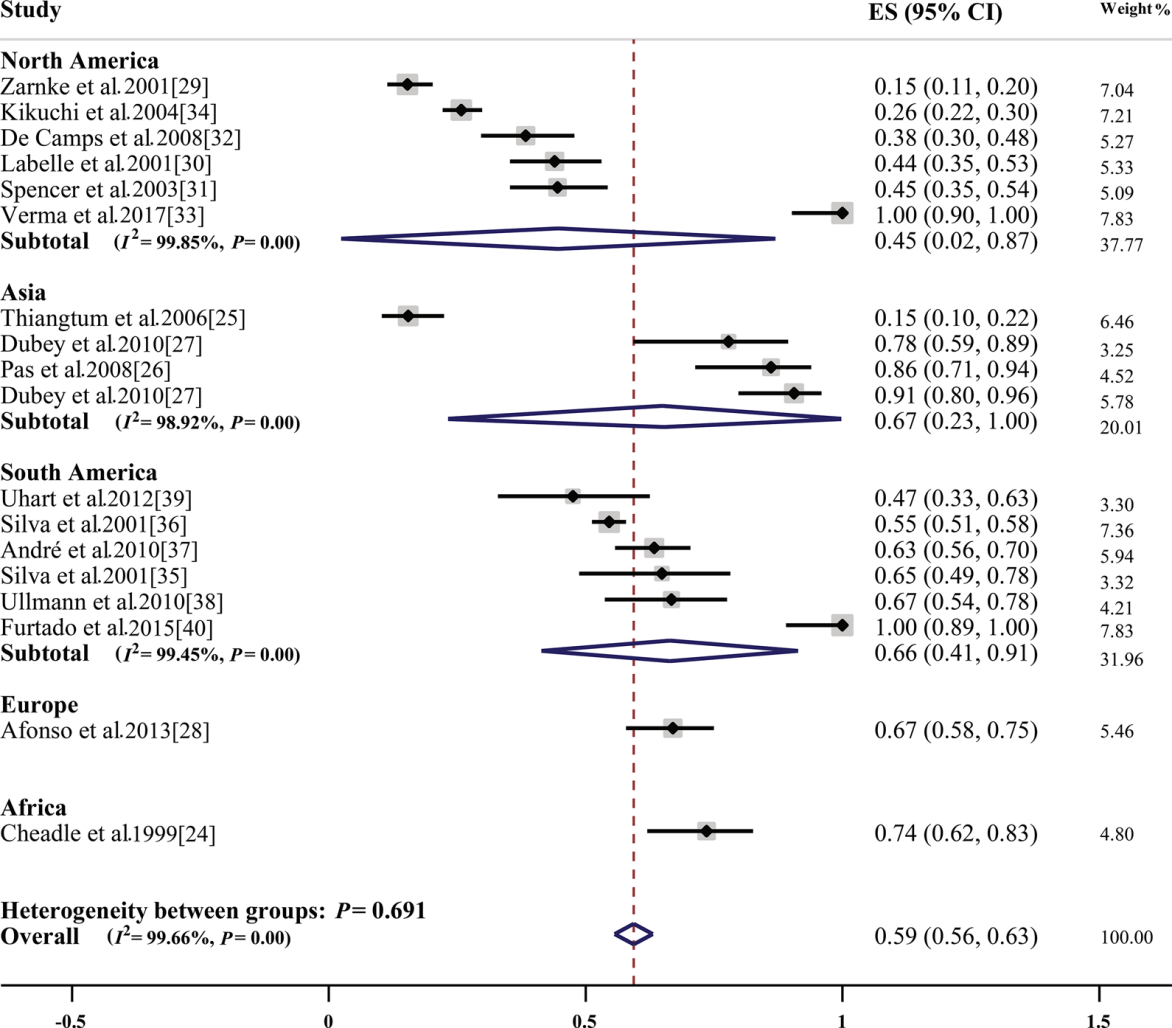
Forest plot for the seroprevalence of *Toxoplasma gondii* in wild Felidae in different countries

The estimates of seroprevalence of *T. gondii* ranged between 0–97% and 15–100% in domestic cats and wild felids, respectively. The global pooled seroprevalence of *T. gondii* reported from 1967 to 2017 was 35% (95% CI: 32–38%) and 59% (95% CI: 56–63%) in domestic cats and wild felids, respectively (Table 2 and Fig. 3). Heterogeneity was substantial, *I*^2^= 99·54%, *P* < 0.001 and *I*^2^ = 99·66%, *P* < 0.001 in domestic cats and wild felids, respectively.

**Table 2:** Global and regional pooled seroprevalence of *Toxoplasma gondii* in domestic cats: results from studies performed in seven continents

Continent	No. of studies	*n*/*N*	Pooled prevalence (95% CI) (%)	Weight	Heterogeneity		
					*df*	*I*^2^ (%)	*P-*value
Global	204	16,722/59,517	35 (32–38)	100	203	99.54	*P* < 0.001
Antarctica	1	141/276	51 (45–57)	0.5	0	–	–
Africa	10	583/1232	51 (20–81)	4.94	9	99.79	*P* < 0.001
Asia	79	4494/22,630	27 (24–30)	39.75	80	98.39	*P* < 0.001
Australia	3	288/443	52 (15–89)	1.46	2	–	–
Europe	45	4706/1116	43 (38–48)	22.04	44	96.88	*P* < 0.001
North America	31	4488/17,232	35 (28–43)	14.64	22	98.61	*P* < 0.001
South America	34	2022/6588	37 (29–45)	16.67	33	99.32	*P* < 0.001

*Abbreviations*: –, impossible to estimate, *df*, degrees of freedom, n, number of positive samples, N, total number of samples

#### Pooled seroprevalence of T. gondii in felids in different continents

The seroprevalence was higher in Australia and Africa where the *T. gondii* seropositivity in domestic cats was 52% (95% CI: 15–89%) and 51% (95% CI: 20–81%), respectively. The lowest seroprevalence estimated was in Asia (27%, 95% CI: 24–30%) (Additional file 2: Table S2). Only one study was performed in Antarctica (Kerguelen Archipelago) and reported a seroprevalence of 51% (95% CI: 45–57%) in 276 feral cats (Additional file 2: Table S2). Significant geographical differences were observed in pooled *T. gondii* seropositivity rates among domestic cats.

In wild felids, the highest pooled seroprevalence was observed in Africa (74%, 95% CI: 62–83%), followed by Asia (67%, 95% CI: 23–100%), Europe (67%, 95% CI: 58–75%) and South America (66%, 95% CI: 41–91%) (Fig. 3).

#### Pooled seroprevalence of T. gondii in male and female felids in different continents

**Fig. 4:**
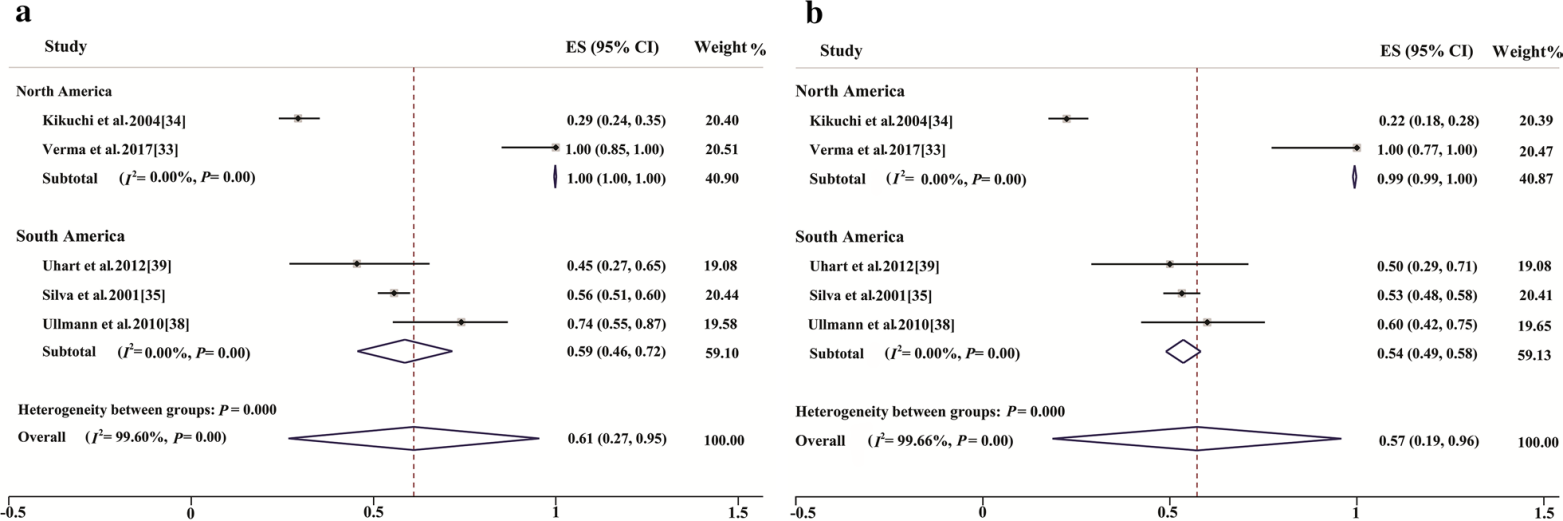
Forest plots for the seroprevalence of *Toxoplasma gondii* in male (**a**) and female (**b**) wild felids

In the subgroup analysis, the global pooled seroprevalence of toxoplasmosis was equal (33%, 95% CI: 29–37%) in male and female domestic cats (Tables 3, 4). The highest pooled seroprevalence in male domestic cats was observed in Australia (62%, 95% CI: 54–70%) followed by Europe (46%, 95% CI: 38–53%) and Africa (43%, 95% CI: 12–75%) (Table 3). Similarly, the highest pooled *T. gondii* seroprevalence in female domestic cats was observed in Australia (68%, 95% CI: 61–75%) followed by Europe (47%, 95% CI: 38–56%) and Africa (45%, 95% CI: 8–82%) (Table 4). The estimates of the global seroprevalence of *T. gondii* in male and female wild felids were 61% (95% CI: 27–95%) and 57% (95% CI: 19–96%), respectively. A forest plot for seroprevalence of *T. gondii* in male and female wild felids in South and North America is provided in Fig. 4.

**Table 3:** Subgroup analysis for comparison of the prevalence of *Toxoplasma gondii* in male domestic cats globally and from different continents

Continent	No. of studies	*n*/*N*	Pooled prevalence (95% CI) (%)	Weight	Heterogeneity		
					*df*	*I*^2^ (%)	*P*-value
Global	101	3169/11,809	33 (29–37)	100	100	98.72	*P* < 0.001
Africa	7	184/445	43 (12–75)	6.96	6	99.33	*P* < 0.001
Asia	44	1164/6127	28 (23–33)	43.64	43	97.54	*P* < 0.001
Australia	2	71/118	62 (54–70)	1.94	1	–	–
Europe	18	1079/2453	46 (38–53)	17.89	17	93.71	*P* < 0.001
North America	10	301/940	33 (24–43)	9.95	19	90.94	*P* < 0.001
South America	20	370/1726	25 (18–32)	19.63	9	96.85	*P* < 0.001

*Abbreviations*: –, impossible to estimate, *df*, degrees of freedom, n, number of positive samples, N, total number of samples

**Table 4:** Subgroup analysis for comparison of the prevalence of *Toxoplasma gondii* in female domestic cats globally and from different continents

Continent	No. of studies	*n*/*N*	Pooled prevalence (95% CI) (%)	Weight	Heterogeneity		
					*df*	*I*^2^(%)	*P*-value
Global	101	3625/13,413	33 (29–37)	100	100	99.00	*P* < 0.001
Africa	7	204/489	45 (8–82)	6.91	6	99.55	*P* < 0.001
Asia	44	1211/6733	27 (22–32)	43.32	43	97.56	*P* < 0.001
Australia	2	86/135	68 (61–75)	1.96	1	–	–
Europe	18	1286/2849	47 (38–56)	18.12	17	96.08	*P* < 0.001
North America	10	335/1039	32 (24–40)	9.90	19	87.51	*P* < 0.001
South America	20	503/2168	28 (20–36)	19.80	9	98.24	*P* < 0.001

*Abbreviations*: –, impossible to estimate, *df*, degrees of freedom, n, number of positive samples, N, total number of samples

### Publication bias

Egger’s regression test revealed that publication bias exerted no significant influence on the overall prevalence of *T. gondii* infection in the cat population (*P* = 0.916).

## Discussion

Only members of the family Felidae (domestic and wild cats) serve as definitive hosts for *T. gondii* worldwide [6]. In this report, we present the first meta-analysis estimating the prevalence of *T. gondii* infection in members of the family Felidae by continent, sex and globally from 1967 to 2017. According to previous reports, the worldwide seroprevalence of *T. gondii* in domestic cats was estimated to be 30–40% [10, 18] and our finding was similar to this range. In the present study, the seroprevalence varied in different areas of the world and we found substantial differences in seroprevalence rates among the different continents.

Accordingly, the *T. gondii* seroprevalence in domestic cats in different continents was in the following order: Australia > Africa > Antarctica > Europe > South America > North America > Asia; although it should be considered that the number of studies for Africa and Australia was relatively low (13 studies in total), and also only one study (276 animals) was included from Antarctica. The lowest seroprevalence was estimated in Asia (27%; 79 studies, containing 81 datasets). Most studies were conducted within countries of Asia. The number of surveys was higher in Brazil (South America; 29 studies).

Overall, our study identified a number of key countries with or without data, emphasizing the need for further studies and more attention to *T. gondii* infection in cats in these countries. Additionally, the prevalence of toxoplasmosis in male and female domestic cats was higher in Australia, Europe and Africa. Moreover, the pooled seroprevalence of *T. gondii* infection in wild felids was higher in males (61%) compared to females (57%). However, Wilking et al. [19] reported that sex might be an important variable in humans; we do not believe it is a significant variable in cats, as under normal conditions, both male and female cats are at equal risk of exposure to *T. gondii* infection sources.

According to the CDC, *T. gondii* accounts for approximately 24% of all estimated deaths due to food-borne pathogens in the USA [20]. *Toxoplasma gondii* infection in food producing animals has become an important public health issue, as a source for human toxoplasmosis by transmission of the parasite *via* pork and wild boar meat and meat products [21, 22]. Cats are the definitive host of *T. gondii* and an infected cat can be a major contributor to environmental contamination. Foroutan et al. [21] and Rostami et al. [22] reported that the presence of cats in the environment was significantly associated with a higher *T. gondii* seropositivity in pigs (19%) and wild boars (23%), respectively, worldwide.

So far, not many investigations have evaluated the prevalence of *T. gondii* in wild felids in the world. As shown in Table 1, the global pooled seroprevalence of *T. gondii* in wild felids was almost 59% from 1967 to 2017 based on 17 studies from 12 countries. This prevalence varies according to different continents, from 45% in North America to 74% in Africa.

Toxoplasmosis is usually more prevalent, especially in moist, warm and low altitude regions [23]. This fact is associated with longer viability of *T. gondii* sporulated oocysts in a warm and humid areas [24]. It should be noted that these data are shown only for the regions which seroprevalence data is reported, as information in many other regions was scarce.

The strength of this investigation includes rigorous methodology, quality assessment, data extraction of included studies, the large sample size of the cats and wild felids included in the meta-analysis, and subgroup analyses considering continents and sex. There are two main limitations in the interpretation of this review. First, there was a lack of studies in some regions from many countries across the world, and many of the available studies suffered from limitations such as the lack of an adequate number of subjects, lack of balanced data on age (as an important item), and non-random sampling of the population at large. Secondly, a potential language bias might exist in our review, as the eligible studies were restricted to papers published in English.

## Conclusions

To the best of our knowledge, this is the first systematic review and meta-analysis providing a general view of the seroprevalence of *T. gondii* infection in members of the family Felidae (domestic and wild cats) from a global perspective. Health education, particularly toward avoiding contact with cats’ feces and integrated preventive control programmes should also be considered.

## Supplementary information

**Additional file 1: Table S1.** PRISMA checklist.

**Additional file 2: Table S2.** Main features of studies regarding the global seroprevalence of *Toxoplasma gondii* in domestic cats.

**Additional file 3: Table S3.** List of countries without data on *Toxoplasma gondii* infection in domestic cats.

**Additional file 4: Table S4.** List of countries without data on *Toxoplasma gondii* infection in wild felids.

## Data Availability

Not applicable.

## Abbreviations

CDC: Centers for Disease Control and Prevention
CI: Confidence interval
IFAT: Immunofluorescence assay test
MAT: Modified agglutination test
PRISMA: Preferred reporting items for systematic reviews and meta-analyses
