# Supplementary material for: The global serological prevalence of Toxoplasma gondii in felids during the last five decades (1967–2017): a systematic review and meta-analysis
Source: Parasit Vectors. 2020 Feb 17;13:82. doi: 10.1186/s13071-020-3954-1 (PMC7026947; doi:10.1186/s13071-020-3954-1)
Supplement: Supplementary file 2 — Additional file 2: Table S2. Main features of studies regarding the global seroprevalence of Toxoplasma gondii in domestic cats. [file 13071_2020_3954_MOESM2_ESM.docx]

**Additional file 2: Table S2.** Main features of studies regarding the global seroprevalence of *Toxoplasma gondii* in domestic cats

| Country | | Publication year |  | Method | Cut-off | Period of sampling | Sampling  season | Sample size | Prevalence (%) | Type of cats | Quality score | Reference |
| --- | --- | --- | --- | --- | --- | --- | --- | --- | --- | --- | --- | --- |
|  | Antarctica | | | | | | | | | | | |
| Kerguelen archipelago | | 2007 |  | MAT | ≥1:40 | 1994-2004 | October 1994-November 2004 | 276 | 51.09 | Feral cats | 10 | [Afonso et al](https://www.ncbi.nlm.nih.gov/pubmed/?term=Afonso%20E%5BAuthor%5D&cauthor=true&cauthor_uid=17672925). [1] |
|  | Africa | | | | | | | | | | | |
| Nigeria | | 1984 |  | SFT | ≥1:10 |  |  | 200 | 94 | Domestic cats | 7 | Arena [2] |
| Egypt | | 2010 |  | MAT | ≥1:5 | 2008-2009 |  | 158 | 97.47 | Feral cats | 7 | Al-Kappany et al. [3] |
| Nigeria | | 2010 |  | LAT | ≥1:64 |  |  | 105 | 36.2 | Owned and stray cats | 8 | Kamani et al. [4] |
| South Africa | | 2012 |  | ELISA | ≥1:64 |  |  | 102 | 7.8 | Cats with a variety of disease conditions | 7 | Lobetti and Lappin [5] |
| Ethiopia | | 2013 |  | MAT | ≥1:25 | 2011 |  | 36 | 91.67 | Feral cats | 7 | Dubey et al. [6] |
| Ethiopia | | 2013 |  | MAT | ≥1:25 | 2011 | Rainy season (July-September) | 48 | 85.4 | Feral cats | 10 | Tiao et al. [7] |
| South Africa | | 2015 |  | IFAT |  | 2013-2014 | October 2013-July 2014 | 159 | 37.1 | Feral cats | 8 | Hammond-Aryee et al. [8] |
| Nigeria | | 2017 |  | MAT | ≥1:20 | 2014 | January-November 2014 | 226 | 4.4 | Domestic cats | 10 | Ayinmode et al. [9] |
| Angola | | 2017 |  | MAT | ≥1:20 | 2014-2016 | May 2014-February 2016 | 102 | 3.9 | Domestic cats (well-cared cats) | 7 | Lopes et al. [10] |
| Algeria | | 2017 |  | MAT | ≥1:6 | 2015 | July-August | 96 | 50 | Stray cats | 10 | Yekkour et al. [11] |
|  | Asia | | | | | | | | | | | |
| Japan | | 1967 |  | HAT | ≥1:64 |  |  | 25 | 60 | Healthy cats |  | Katsube et al. [12] |
| Japan | | 1972 |  | IFAT | ≥1:8 |  |  | 90 | 44.44 | Domestic cats | 8 | Werner and Walton [13] |
| Taiwan | | 1975 |  | IHA or MBDT |  |  |  | 58 | 22.4 | Domestic pet cats | 6 | Durfee et al. [14] |
| Indonesia | | 1976 |  | IHA |  |  |  | 69 | 40.6 | Cats | 6 | Durfee et al. [15] |
| Georgia | | 1979 |  | DT | ≥1:2 | 1977 |  | 39 | 23.08 | Feral and pet cats | 9 | Teutsch et al. [16] |
| Japan | | 1981 |  | LAT | ≥1:64 | 1979-1980 |  | 1539 | 13.3 | House cats | 6 | Ohshima et al. [17] |
| Northern India | | 1985 |  | IHA |  |  |  | 80 | 33.7 | Cats | 6 | Chhabra et al. [18] |
| Lebanon | | 1985 |  | IFAT | ≥1:64 | 1980-1983 | September 1980-July 1983 | 324 | 69.75 | Domestic stray and owned cats | 8 | Deeb et al. [19] |
| Georgia | | 1989 |  | ELISA | ≥1:64 |  |  | 188 | 41 | Healthy and clinically ill cats | 7 | Lappin et al. [20] |
| Taiwan | | 1990 |  | ELISA |  |  |  | 117 | 7.7 | Feral cats | 6 | Lin et al. [21] |
| China | | 1990 |  | IHA | ≥1:64 |  |  | 47 | 2.1 | Cats | 7 | Shen et al. [22] |
| Taiwan | | 1998 |  | ELISA | ≥0.02 | 1995-1996 |  | 157 | 28.66 | Stray cats and pet cats | 9 | Lin et al. [23] |
| Japan | | 1998 |  | LAT | ≥1:64 | 1994-1995 | May1994-June1995 | 471 | 8.7 | Pet cats | 10 | Maruyama et al. [24] |
| Japan | | 1998 |  | LAT | ≥1:64 | 1997 |  | 800 | 6 | Domiciled cats | 10 | Nogami et al. [25] |
| Korea | | 1999 |  | ELISA | OD >0.275 | 1996-1998 |  | 198 | 17.68 | Stray cat | 8 | Sohn and Nam [26] |
| Japan | | 2001 |  | ELISA | OD >0.1 |  |  | 193 | 20.7 | Domestic cats | 6 | Kimbita et al. [27] |
| Japan | | 2002 |  | ELISA | OD ≥0.2 | |  | 192 | 21.9 | Domestic cats | 6 | Huang et al. [28] |
| Japan | | 2003 |  | LAT | ≥1:64 | 1994-1999 |  | 1447 | 5.4 | Pet cats | 10 | Maruyama et al. [29] |
| Thailand | | 2003 |  | SFT | ≥1:16 |  |  | 315 | 7.3 | Households and temples cats | 8 | Sukthana et al. [30] |
| Japan | | 2004 |  | ELISA | OD ≥0.2 | |  | 179 | 20.1 | Domestic cats | 6 | Huang et al. [31] |
| Israel | | 2004 |  | ELISA | ≥1:80 | 1999-2000 | April 1999-April 2000 | 1062 | 16.8 | Stray, domestic, healthy and clinically ill cats | 10 | Salant and Spira [32] |
| Iran | | 2006 |  | IFAT | ≥1:32 | 2002 | April-September | 100 | 63 | Stray and household cats | 10 | Haddadzadeh et al. [33] |
| Pakistan | | 2006 |  | LAT | ≥1:16 |  |  | 50 | 56 | Stray and domestic cats | 10 | Shahzad et al. [34] |
| China | | 2007 |  | MAT | ≥1:40 | 2006 | January-September | 34 | 79.4 | Cats of local breeds | 8 | Dubey et al. [35] |
| Iran | | 2007 |  | IFAT | ≥1:20 | 2004-2005 | June 2004-April 2005 | 50 | 86 | Domestic cats | 8 | [Hooshyar et al](https://scholar.google.com/citations?user=Uhc9ziIAAAAJ&hl=en&oi=sra). [36] |
| Thailand | | 2007 |  | LAT | ≥1:64 | 2001-2002 | October 2001-September 2002 | 592 | 11 | Stray cats | 9 | Jittapalapong et al. [37] |
| Malaysia | | 2008 |  | IFAT | ≥1:200 |  |  | 55 | 14.55 | Stray cats | 6 | Chandrawathani et al. [38] |
| [Turkey](https://www.tandfonline.com/doi/abs/10.4081/ijas.2008.113) | | 2008 |  | SFT | ≥1:16 | 2003 |  | 72 | 76.4 | Stray cats | 10 | Karatepe et al. [39] |
| Korea | | 2008 |  | ELISA | OD >0.25 | 2007 | April-October | 174 | 16.1 | Stray cats | 8 | Kim et al. [40] |
| Turkey | | 2008 |  | IFAT | ≥1:16 |  |  | 99 | 34.3 | Cats | 8 | [Özkan et al](https://scholar.google.com/citations?user=cijAu7IAAAAJ&hl=en&oi=sra). [41] |
| China | | 2008 |  | ELISA or LAT |  | 1999-2005 |  | 335 | 14.9 | Pet cats | 8 | Yu et al. [42] |
| Iran | | 2009 |  | LAT | ≥1:1 | 2004 | April-November | 100 | 40 | Stray cats | 10 | Sharif et al. [43] |
| China | | 2009 |  | ELISA | IRPC >2.1 |  |  | 206 | 25.24 | Stray and household cats | 8 | Zhang et al. [44] |
| Philippines | | 2010 |  | LAT | Titre ≥15 IU/mL |  |  | 60 | 46.67 | Healthy cats, kept outdoors with free access to outside sources | 8 | Advincula et al. [45] |
| Iran | | 2010 |  | MAT | ≥1:20 | 2008 | February–August | 140 | 32.1 | Owned and stray cats | 9 | Akhtardanesh et al. [46] |
| Iran | | 2010 |  | ELISA | ≥1:64 | 2006-2007 | March 2006-February 2007 | 50 | 8 | Stray cats | 8 | Javadi et al. [47] |
| Thailand | | 2010 |  | SFT | ≥1:16 | 2006 | March-May 2006 | 1490 | 4.8 | Owned and stray cats | 10 | Jittapalapong et al. [48] |
| Korea | | 2010 |  | ELISA | OD >0.25 | 2008 | April-September | 72 | 15.3 | Stray cats | 10 | Lee et al. [49] |
| Iraq | | 2010 |  | LAT | ≥1:2 | 2006-2007 | November 2006-April 2007 | 90 | 33.33 | Stray (Urban and rural) cats | 8 | Al-Ramahi et al. [50] |
| Iran | | 2011 |  | ICHA |  | 2006-2009 | December 2006-November 2009 | 198 | 24.75 | Companion cats | 10 | Mosallanejad et al. [51] |
| Saudi Arabia | | 2011 |  | ELISA |  |  |  | 156 | 62.8 | Household and stray cats | 8 | [Al-Mohammed](https://scholar.google.com/citations?user=_wMd6cAAAAAJ&hl=en&oi=sra)  [52] |
| Korea | | 2011 |  | ELISA |  | 2009-2010 |  | 182 | 12.64 | Household and stray cats | 8 | Chong et al. [53] |
| Iran | | 2011 |  | MAT | ≥1:25 |  |  | 100 | 54 | Stray and household cats | 6 | Hamidinejat et al. [54] |
| Sri Lanka | | 2011 |  | MAT | ≥1:25 | 2008 | January-April 2008 | 86 | 30.2 | Companion cats | 6 | Kulasena et al. [55] |
| Korea | | 2011 |  | ELISA | OD >0.25 | 2008 | March-November 2008 | 456 | 14.3 | Feral cats | 10 | Lee et al. [56] |
| Iran | | 2011 |  | MAT | ≥1:20 | 2008-2010 | October 2008-January 2010 | 130 | 35.38 | Stray and household cats | 10 | [Raeghi et al](https://scholar.google.com/citations?user=pKJt2aAAAAAJ&hl=en&oi=sra). [57] |
| China | | 2011 |  | MAT | ≥1:25 | 2010-2011 | November 2010-July 2011 | 221 | 21.3 | Household and stray cats | 10 | Wu et al. [58] |
| China | | 2012 |  | MAT | ≥1:20 | 2009-2010 | November 2009-September 2010 | 64 | 57.8 | Stray cats | 9 | Qian et al. [59] |
| Thailand | | 2012 |  | MAT | ≥1:25 | 2009 | May-July | 348 | 10.1 | Pet cats | 10 | Sukhumavasi et al. [60] |
| China | | 2012 |  | ELISA |  | 2010-2011 | January 2010-December 2011 | 145 | 11.7 | Unwanted and stray cats | 8 | Wang et al. [61] |
| China | | 2012 |  | ELISA |  | 2010-2011 | May 2010-April 2011 | 263 | 20.15 | Pet cats | 8 | Zhu et al. [62] |
| China | | 2012 |  | ELISA |  | 2010-2011 | May 2010-April 2011 | 45 | 35.6 | Pet cats | 8 | Zhu et al. [62] |
| Kuwait | | 2013 |  | IHA | ≥1:80 | 2011-2012 | June 2011-May 2012 | 240 | 19.6 | Cats within dairy farms | 8 | Abdou et al. [63] |
| Thailand | | 2013 |  | LAT | ≥1:64 | 2006-2007 | June 2006-September 2007 | 36 | 8.3 | Domestic cats | 8 | Arunvipas et al. [64] |
| Taiwan | | 2013 |  | ELISA | ≥0.02 | 2008-2010 |  | 100 | 12 | Pet cats | 8 | Fuh et al. [65] |
| Korea | | 2013 |  | ELISA | OD >0.25 | 2009-2011 | April 2009-June 2011 | 437 | 2.2 | Clinically healthy household cats | 9 | Hong et al. [66] |
| Philippines | | 2013 |  | ELISA | ≥1:32 |  |  | 30 | 46.67 | Domestic pets | 8 | Reyes et al. [67] |
| Iraq | | 2013 |  | LAT | ≥1:32 | 2008 | February-December 2008 | 207 | 30.4 | Stray cats | 10 | Switzer et al. [68] |
| Pakistan | | 2014 |  | ELISA |  | 2012 | January-December 2012 | 420 | 23.33 | Domestic cats | 8 | Ahmad and Qayyum. [69] |
| Turkey | | 2014 |  | ELISA | ≥1:16 |  |  | 1121 | 35.6 | Stray cats (healthy and deceased cats) | 6 | [Can et al](https://scholar.google.com/citations?user=zKTwlAEAAAAJ&hl=fa&oi=sra). [70] |
| Iran | | 2014 |  | IFAT | ≥1:16 |  |  | 108 | 2.7 | Feral and stray cats | 6 | Derakhshan and mousavi [71] |
| China | | 2014 |  | ELISA | IRPC >2.1 | 2013 | Janurary-December 2013 | 116 | 20.7 | Pet cats | 9 | Liu et al. [72] |
| Korea | | 2014 |  | ELISA |  |  |  | 118 | 5.93 | Stray cats | 8 | Park et al. [73] |
| China | | 2015 |  | ELISA |  | 2013-2014 |  | 97 | 25.8 | Free-roaming cats | 6 | Jiang et al. [74] |
| China | | 2015 |  | ELISA |  | 2010 | November-December 2010 | 185 | 21.6 | Household and stray cats | 10 | Cai et al. [75] |
| Japan | | 2015 |  | LAT | ≥1:64 | 1999–2001 | April 1999-March 2001 | 233 | 5.6 | Shelter cats | 10 | Oi et al. [76] |
| Japan | | 2015 |  | LAT | ≥1:64 | 2009–2011 | April 2009-March 2011 | 104 | 6.7 | Shelter cats | 10 | Oi et al. [76] |
| Iran | | 2015 |  | ELISA | ISR >1.10 | 2007-2008 | December 2007-August 2008 | 107 | 64.48 | Stray cats | 10 | [Tehrani-sharif et al](https://scholar.google.com/citations?user=Jbsj3YgAAAAJ&hl=en&oi=sra). [77] |
| China | | 2015 |  | MAT | ≥1:25 | 2014 |  | 42 | 50 | Pet, stray and farm cats | 9 | Yang et al. [78] |
| Russia | | 2016 |  | EIA |  |  |  | 61 | 14.7 | Domestic cats | 10 | Pavlova et al. [79] |
| China | | 2016 |  | MAT | ≥1:25 | 2014-2015 | March 2014-May 2015 | 362 | 19.34 | Stray and pet cats | 9 | Cong et al. [80] |
| Turkey | | 2016 |  | SFT | ≥1:16 |  |  | 102 | 44.1 | Stray and companion cats | 8 | Erkılıç et al. [81] |
| China | | 2016 |  | IHA | ≥1:64 | 2012-2015 | May 2012-August 2015 | 1141 | 15.43 | Stray and companion cats | 10 | Kang et al. [82] |
| China | | 2017 |  | MAT | ≥1:25 | 2015-2017 |  | 28 | 7.1 | Pet cats | 7 | Yang et al. [83] |
| Japan | | 2017 |  | LAT | ≥1:32 |  |  | 419 | 17.4 | Cats visiting animal hospitals | 8 | Abdelbaset et al. [84] |
| Qatar | | 2017 |  | MAT | ≥1:25 | 2014-2015 | September 2014-September 2015 | 495 | 82 | Stray cats | 10 | Boughattas et al. [85] |
| Korea | | 2017 |  | ELISA |  | 2013-2015 | Summers of 2013 and 2015 | 112 | 50 | Feral cats | 9 | Hwang et al. [86] |
| Korea | | 2017 |  | ELISA | OD ≥0.4 |  |  | 150 | 4 | Household and feral cats | 8 | Kim et al. [87] |
| Japan | | 2017 |  | GLIPS | 5.0 × 10000 LU | 2013-2017 |  | 1363 | 9 | Free-ranging and feral cats | 9 | Matsuu et al. [88] |
| Iran | | 2017 |  | ELISA |  | 2013 | February-December 2013 | 100 | 30 | Stray cats | 8 | Mosallanejad et al. [89] |
| China | | 2017 |  | ELISA | IRPC >2.1 | 2015-2016 | March 2015-May 2016 | 843 | 21.12 | Domestic (urban and rural) cats | 10 | [Wang et al](https://scholar.google.com/citations?user=C4rJ3IAAAAAJ&hl=en&oi=sra). [90] |
|  | Australia | | | | | | | | | | | |
| Western Australia | | 1983 |  | IHA |  | 1979 |  | 74 | 32.43 | Feral and domestic cats | 6 | Jakob‐Hoff and Dunsmore [91] |
| Australia | | 1999 |  | ELISA | OD ≥0.5 |  |  | 103 | 38.8 | Domestic cats | 8 | Sumner and Ackland [92] |
| Australia | | 2014 |  | MAT | ≥1:64 | 2009-2013 |  | 266 | 84.2 | Feral and stray cats | 10 | Fancourt [93] |
|  | Europe | | | | | | | | | | | |
| Norway | | 1978 |  | SFT | ≥1:8 |  |  | 87 | 24.1 | Domestic cats | 6 | Kapperud [94] |
| England | | 1987 |  | Serology |  |  |  | 51 | 47 | Farm cats | 6 | Gethings et al. [95] |
| [Scotland](https://en.wikipedia.org/wiki/Scotland) | | 1987 |  | SFT | ≥1:10 |  |  | 158 | 19 | Stray and domiciled cats | 10 | Jackson et al. [96] |
| Sweden | | 1990 |  | ELISA |  | 1986-1987 |  | 244 | 41.8 | Pet cats | 7 | [Uggla et al](https://www.ncbi.nlm.nih.gov/pubmed/?term=Uggla%20A%5BAuthor%5D&cauthor=true&cauthor_uid=2260515). [97] |
| Germany | | 1994 |  | ELISA |  |  |  | 306 | 45.1 | Cats kept indoors and stray cats | 6 | [Tenter et al](https://www.ncbi.nlm.nih.gov/pubmed/?term=Tenter%20AM%5BAuthor%5D&cauthor=true&cauthor_uid=8058366). [98] |
| UK | | 1996 |  | HAT |  | 1989 | Winter 1989 | 45 | 62.2 | Feral cats | 8 | [Yamaguchi et al](https://scholar.google.com/citations?user=ls6t0JYAAAAJ&hl=en&oi=sra). [99] |
| Italy | | 1997 |  | ELISA | ≥1:64 |  |  | 490 | 33.26 | Stray cats |  | Damore et al. [100] |
| Czech Republic | | 1998 |  | IFAT | ≥1:10 | 1995-1997 | November 1995-June 1997 | 390 | 61.5 | ill cats | 10 | Svobodova et al. [101] |
| Belgium | | 2002 |  | DMA | ≥1:40 | 1998-2000 | October 1998-February 2000 | 346 | 70.2 | Urban stray cats | 10 | Dorny et al. [102] |
| Poland | | 2002 |  | IFAT |  |  |  | 200 | 52.5 | Homeless, shelters and pet cats visiting outpatients clinics | 6 | Smielewska-Loś and Pacoń [103] |
| Spain | | 2003 |  | MAT | ≥1:25 |  |  | 220 | 45 | Feral and domiciled cats | 8 | Gauss et al. [104] |
| Italy | | 2004 |  | IFAT |  | 2000-2001 |  | 54 | 37 | Domestic cats | 8 | Porqueddu et al. [105] |
| Spain | | 2004 |  | IFAT | ≥1:80 |  |  | 585 | 32.3 | Household, stray and farm cats | 8 | Miro´ et al. [106] |
| Italy | | 2006 |  | DAT | ≥1:40 | 2003-2004 | January 2003-December 2004 | 573 | 40.7 | Stray cats | 10 | Papini et al. [107] |
| France | | 2006 |  | MAT | ≥1:40 | 1993-2004 | January 1993-October 2004 | 301 | 18.6 | Domestic cats | 8 | Afonso et al. [108] |
| France | | 2006 |  | DAT | ≥1:64 | 2001-2003 | June 2001-June 2003 | 50 | 40 | Domestic cats with uveitis and systemic disease | 8 | Meunier et al. [109] |
| Czech Republic | | 2006 |  | IFAT | ≥1:40 | 2002-2006 |  | 286 | 44.1 | Pet cats | 8 | Sedlak and Bartova [110] |
| Italy | | 2007 |  | IHA | ≥5 IU/ml | 2005-2006 | July 2005-February 2006 | 189 | 69.3 | Stray cats | 10 | Natale et al. [111] |
| Poland | | 2007 |  | DAT | ≥1:32 ≥1:40 | |  | 53 | 50.9 | Farm cats | 6 | Sroka et al. [112] |
| Hungary | | 2008 |  | IFAT | ≥1:20 |  |  | 330 | 47.6 | Cats |  | Hornok et al. [113] |
| Portugal | | 2008 |  | MAT | ≥1:20 | 2004-2005 | May 2004-May 2005 | 204 | 35.8 | Domestic cats | 10 | Lopes et al. [114] |
| Spain | | 2008 |  | IFAT | >1:80 |  |  | 592 | 17.39 | Strays, farms and household cats | 9 | Montoya et al. [115] |
| Italy | | 2009 |  | MAT | ≥1:20 |  |  | 115 | 38.3 | Cats | 6 | Macrì et al. [116] |
| Spain | | 2009 |  | MAT | ≥1:25 | 2008 | July-November | 59 | 84.7 | Feral cats | 10 | Milla´n et al. [117] |
| France | | 2010 |  | MAT | ≥1:40 | 1991-2005 |  | 861 | 52.7 | Domeastic cats | 8 | [Afonso et al](https://scholar.google.com/citations?user=4I7Qy4MAAAAJ&hl=fa&oi=sra). [118] |
| Portugal | | 2010 |  | DAT | ≥1:80 | 2003-2005 | November 2003-July 2005 | 194 | 24.2 | Stray cats | 9 | Duarte et al. [119] |
| Ireland | | 2010 |  | ELISA | ≥1:64 | 2008 | January-May | 83 | 21.7 | Client-owned and stray cats | 10 | Juvet et al. [120] |
| Italy | | 2010 |  | MAT | ≥1:20 |  |  | 50 | 40 | Domestic cats | 7 | Mancianti et al. [121] |
| Poland | | 2010 |  | DAT | ≥1:40 |  |  | 135 | 65.9 | Domeastic cats | 6 | Michalski et al. [122] |
| Scotland | | 2011 |  | ELISA | ≥1:64 | 2009 | June-August | 52 | 11.5 | Stray and household cats | 8 | Bennett et al. [123] |
| Western Romania | | 2011 |  | ELISA | S/P values >50% | 2008-2009 |  | 173 | 66.5 | Feral cats | 10 | [Darabus et al](https://scholar.google.com/citations?user=GVGCytYAAAAJ&hl=en&oi=sra). [124] |
| Netherlands | | 2011 |  | ELISA | S/P value ≥50% | 2007–2010 |  | 236 | 47 | Household cats | 9 | Györke et al. [125] |
| Finland | | 2012 |  | DAT | ≥1:40 | 2008-2009 | September 2008-August 2009 | 490 | 48.4 | Purebred pet and shelter cats | 10 | Jokelainen et al. [126] |
| Netherlands | | 2012 |  | IELISA |  | 2005-2010 | May 2005-August 2010 | 450 | 20.2 | Domestic cats | 9 | Opsteegh et al. [127] |
| Italy | | 2012 |  | IFAT | ≥1:64 | 2008-2010 | January 2008-January 2010 | 203 | 30.5 | Stray cats | 8 | [Spada et al](https://scholar.google.com/citations?user=WSjqmEEAAAAJ&hl=en&oi=sra). [128] |
| Portugal | | 2012 |  | DAT | ≥1:20 | 2009-2010 | June 2009-April 2010 | 423 | 44.2 | Stray cats | 10 | Waap et al. [129] |
| France | | 2013 |  | MAT | ≥1:48 | 1996-2006 |  | 29 | 65.5 | Domestic cats | 10 | Afonso et al. [130] |
| Latvia | | 2013 |  | IELISA |  | 2011-2012 | November 2011-February 2012 | 242 | 51.7 | Pet cats | 10 | Deksne et al. [131] |
| Italy | | 2013 |  | IFAT | ≥1:64 | 2008-2010 |  | 78 | 21.8 | Stray cats | 8 | Spada et al. [132] |
| Portugal | | 2014 |  | MAT | ≥1:40 | 2007-2008 | October 2007-March 2008 | 215 | 20.5 | Domestic cats | 10 | Esteves et al. [133] |
| Albania | | 2014 |  | IFAT | ≥1:100 | 2008-2010 |  | 146 | 62.3 | Domestic cats | 10 | Silaghi et al. [134] |
| Estonia | | 2015 |  | DAT | ≥1:40 | 2013 | January-December 2013 | 490 | 60.8 | Pet and shelter cats | 8 | Must et al. [135] |
| Norway | | 2015 |  | DAT | ≥1:40 | 2009 |  | 478 | 41 | Pet and domestic pedigree cats | 10 | Sævik et al. [136] |
| Italy | | 2016 |  | IFAT | ≥1:64 | 2014 | June-December | 82 | 29.3 | Stray cats | 8 | Spada et al. [137] |
| Italy | | 2017 |  | IFAT | ≥1:64 | 2014-2015 | September 2014-December 2015 | 78 | 42.3 | Privately owned cats | 8 | Veronesi et al. [138] |
|  | North America | | | | | | | | | | | |
| Canada | | 1978 |  | SFT | ≥1:16 |  |  | 152 | 19.7 | Cats | 6 | Tizard et al. [139] |
| USA (Washington) | | 1982 |  | DT |  |  |  | 87 | 31 | Pound-source cats | 6 | Ladiges et al. [140] |
| USA (Oklahoma) | | 1990 |  | LAT | ≥1:16 | 1987-1988 | July 1987 - June 1988 | 618 | 22.17 | Clinically ill and clinically healthy cats and cats with unknown health status | 10 | Rodgers and Baldwin [141] |
| USA (Iowa) | | 1992 |  | MAT | ≥1:32 |  |  | 74 | 41.9 | Domestic cats | 8 | Smith et al. [142] |
| Panama | | 1995 |  | DAT |  |  |  | 241 | 45.6 | Pet cats | 9 | Frenkel et al. [143] |
| USA (Colorado) | | 2000 |  | ELISA | >1:32 | 1993-1995 | May 1993-August 1995 | 206 | 23.3 | Client-owned and shelter cats | 8 | Hill et al. [144] |
| USA  (Rhode Island) | | 2002 |  | MAT | ≥1:25 | 1998 | November-December | 200 | 42 | Stray and client-owned cats | 10 | [DeFeo et al](https://www.ncbi.nlm.nih.gov/pubmed/?term=DeFeo%20ML%5BAuthor%5D&cauthor=true&cauthor_uid=12492287). [145] |
| USA (Ohio) | | 2002 |  | MAT | ≥1:25 |  |  | 275 | 48.4 | Domestic cats | 8 | Dubey et al. [146] |
| USA (Michigan) | | 2002 |  | IFAT | ≥1:40 | 1999-2001 | February 1999-April 2001 | 196 | 24.49 | Domestic cats | 8 | Rossano et al. [147] |
| USA (North Carolina) | | 2004 |  | MAT | ≥1:25 |  |  | 176 | 50.6 | Feral and pet domestic cats | 8 | [Nutter et al](https://scholar.google.com/citations?user=jDHDttwAAAAJ&hl=en&oi=sra). [148] |
| USA | | 2005 |  | ELISA | ≥1:64 | 1998-2001 | May 1998-December 2001 | 12628 | 22.5 | Clinically ill and client-owned cats | 8 | Vollaire et al. [149] |
| Guatemala | | 2005 |  | MAT | ≥1:32 | 2001 | July2001 | 30 | 53.3 | Domestic cats | 10 | Lickey et al. [150] |
| Grenada | | 2006 |  | MAT | ≥1:25 |  |  | 40 | 35 | Domestic cats | 6 | Asthana et al. [151] |
| Mexico | | 2007 |  | IELISA | ≥0.32 |  |  | 80 | 28.75 | Stray Cats | 8 | García-Márquez et al. [152] |
| Mexico | | 2007 |  | MAT | ≥1:20 | 2006 | August-November 2006 | 105 | 21 | Domestic cats | 10 | Alvarado-Esquivel et al. [153] |
| USA (California) | | 2007 |  | ELISA | ≥1:64 | 2003-2005 |  | 123 | 15.4 | Feral cats | 7 | Dabritz et al. [154] |
| USA (Hawaii) | | 2007 |  | ELISA | ≥1:64 | 2002-2004 | April 2002-May 2004 | 67 | 34.3 | Domestic cats | 8 | [Danner et al](https://scholar.google.com/citations?user=0_DzR64AAAAJ&hl=en&oi=sra). [155] |
| St. Kitts | | 2007 |  | MAT | ≥1:20 | 2005-2006 |  | 106 | 84.9 | Stray cats | 7 | Moura et al. [156] |
| Midwestern United States | | 2008 |  | MAT | ≥1:25 | 2003-2005 | January 2003-June 2005 | 34 | 29.4 | Feral domestic cats | 10 | De Camps et al. [157] |
| Mexico | | 2008 |  | IELISA |  |  |  | 169 | 21.9 | Domestic (client-owned) cats | 8 | Besne´-Merida et al. [158] |
| Grenada | | 2009 |  | MAT | ≥1:25 | 2004-2007 | June 2004-December 2007 | 176 | 29 | Pet and Feral cats | 10 | Dubey et al. [159] |
| St Kitts | | 2009 |  | MAT | ≥1:10 | 2005-2006 |  | 96 | 73.9 | Feral cats | 8 | [Dubey et al](https://www.ncbi.nlm.nih.gov/pubmed/?term=Dubey%20JP%5BAuthor%5D&cauthor=true&cauthor_uid=19402949). [160] |
| USA (Pennsylvania) | | 2009 |  | MAT | ≥1:25 | 2008 | January-July | 210 | 19.5 | Unwanted stray cats | 10 | [Dubey et al](https://www.ncbi.nlm.nih.gov/pubmed/?term=Dubey%20JP%5BAuthor%5D&cauthor=true&cauthor_uid=19402949). [161] |
| Mexico | | 2009 |  | MAT | ≥1:25 | 2007 | April-September 2007 | 150 | 9.3 | Stray and pet cats | 10 | [Dubey et al](https://www.ncbi.nlm.nih.gov/pubmed/?term=Dubey%20JP%5BAuthor%5D&cauthor=true&cauthor_uid=19402949). [162] |
| USA | | 2010 |  | ELISA | ≥1:64 | 2003-2004 | January 2003-January 2004 | 123 | 13 | Healthy cats and cats with endogenous uveitis | 10 | Powell et al. [163] |
| USA (Virginia) | | 2011 |  | IFAT | ≥1:25 | 2008-2010 | September 2008-May 2010 | 232 | 27.2 |  | 8 | Hsu et al. [164] |
| Mexico | | 2012 |  | IELISA | Percent positivity (PP) ≥15% |  |  | 220 | 91.8 | Domestic cats | 6 | Castillo-Morales et al. [165] |
| Mexico | | 2013 |  | IELISA |  |  |  | 50 | 100 | Stray cats | 6 | Jimenez-Coello et al. [166] |
| USA (Ohio) | | 2015 |  | MAT | ≥1:25 |  |  | 200 | 51.5 | Cats with sporotrichosis | 10 | Ballash et al. [167] |
| Mexico | | 2015 |  | IELISA |  | 2008 | February-July | 48 | 29.2 | Unwanted and stray cats | 7 | [Rico-Torres et al](https://www.ncbi.nlm.nih.gov/pubmed/?term=Rico-Torres%20CP%5BAuthor%5D&cauthor=true&cauthor_uid=25737051). [168] |
| Panama | | 2017 |  | IELISA | OD >0.35 | 2015–2016 | October 2015–October 2016 | 120 | 25 | Domestic pets | 8 | Rengifo-Herrera et al. [169] |
|  | South America | | | | | | | | | | | |
| Argentina | | 1995 |  | IHA | ≥1:32 | 1993 |  | 169 | 19.5 | Pet cats | 9 | Fernández et al. [170] |
| Brazil | | 1999 |  | IFAT | ≥1:16 | 1996-1997 | February 1996-January 1997 | 248 | 17.7 | Domestic outpatient cats (Outdoor and Indoor cats) | 10 | Lucas et al. [171] |
| Brazil | | 2002 |  | MAT | ≥1:20 | 1993-2000 |  | 502 | 26.3 | Domestic cats | 10 | Silva et al. [172] |
| Brazil | | 2004 |  | MAT | ≥1:20 | 2003-2004 |  | 58 | 84.5 | Domestic cats | 10 | Dubey et al. [173] |
| Brazil | | 2004 |  | ELISA |  | 1999–2000 |  | 100 | 40 | Stray cats | 8 | Meireles et al. [174] |
| Brazil | | 2006 |  | MAT | ≥1:25 | 2002 | May-October 2002 | 63 | 87.3 | Cats | 8 | Cavalcante et al. [175] |
| Colombia | | 2006 |  | MAT | ≥1:5 | 2005 | May-November | 170 | 45.3 | Cats | 10 | Dubey et al. [176] |
| Brazil | | 2006 |  | MAT | ≥1:25 | 2003 |  | 237 | 35.4 | Stray cats | 10 | Pena et al. [177] |
| Brazil | | 2007 |  | IFAT | ≥1:64 |  |  | 400 | 25 | Domestic cats | 8 | [Bresciani et al](https://scholar.google.com/citations?user=WllffSYAAAAJ&hl=en&oi=sra). [178] |
| Brazil | | 2007 |  | IHA | ≥1:16 | 2002 | June-August | 38 | 92.1 | Domestic, unowned and owned cats |  | Mendes-de-Almeida et al. [179] |
| Brazil | | 2010 |  | ELISA | OD >0.221 |  |  | 59 | 78 | Stray and household cats | 6 | [Duarte et al](https://scholar.google.com/citations?user=R84Hk-wAAAAJ&hl=en&oi=sra). [119] |
| Brazil | | 2010 |  | IFAT | ≥1:64 | 2008-2009 | August 2008-July 2009 | 300 | 14.33 | Domestic cats | 8 | Rosa et al. [180] |
| Argentina | | 2011 |  | IFAT | ≥1:25 | 2007-2008 | December 2007-December 2008 | 513 | 22.6 | Domestic (public places and households) cats | 10 | Lopez et al. [181] |
| Brazil | | 2011 |  | IFAT | ≥1:64 | 2007-2009 |  | 70 | 15.7 | Domestic cats | 10 | Coelho et al. [182] |
| Brazil | | 2011 |  | IFAT | ≥1:16 | 2007 |  | 282 | 16.3 | Cats | 8 | Cruz et al. [183] |
| Brazil | | 2012 |  | IFAT | ≥1:40 | 2008-2009 | October 2008-January 2009 | 200 | 50.5 | Cats with outdoor access (Peridomestic cats) | 8 | Braga et al. [184] |
| Brazil | | 2012 |  | IFAT or MAT | ≥1:16 or ≥1:25 | 2007-2010 | July 2007-May 2010 | 118 | 59.3 | Feral and domestic cats | 10 | Costa et al. [185] |
| Brazil | | 2012 |  | IFAT | ≥1:16 |  |  | 251 | 20.32 | Asymptomatic and symptomatic | 8 | Sobrinho et al. [186] |
| Brazil | | 2013 |  | IFAT | ≥1:64 | 2010 | March-October | 386 | 16.3 | Unwanted or stray cats | 10 | Cardia et al. [187] |
| Brazil | | 2014 |  | MAT | ≥1:25 | 2012-2013 |  | 53 | 52.8 | Domestic cats (domiciled and stray cats) | 10 | Fournier et al. [188] |
| Brazil | | 2014 |  | IHA | ≥1:16 | 2009-2010 | May 2009-August 2010 | 108 | 5.6 | Stray, healthy and deceased cats | 10 | Bastos et al. [189] |
| Peru | | 2014 |  | IHA |  | 2011 |  | 154 | 9.7 | Stray cats | 8 | Cerro et al. [190] |
| Brazil | | 2014 |  | IFAT | ≥1:16 | 2011 |  | 201 | 43.8 | Domestic cats | 9 | Feitosa et al. [191] |
| Brazil | | 2014 |  | IFAT | ≥1:40 | 2013 | January-April 2013 | 151 | 32.5 | Domestic cats | 10 | Sousa et al. [192] |
| Brazil | | 2015 |  | IFAT | ≥1:64 | 2007-2011 | November 2007-February 2011 | 213 | 6.6 | Cats with sporotrichosis | 10 | Barros et al. [193] |
| Brazil | | 2015 |  | IFAT | ≥1:16 | 2000-2010 | February 2000-January 2010 | 29 | 82.76 | Domestic cats | 8 | Furtado et al. [194] |
| Chile | | 2015 |  | ELISA |  |  |  | 60 | 48.3 | Domestic (indoor and outdoor) cats | 8 | Toro et al. [195] |
| Brazil | | 2015 |  | IFAT | ≥1:64 | 2012-2013 | November 2012-October 2013 | 89 | 24.7 | Domestic cats | 10 | Figueiredo de Souza et al. [196] |
| Brazil | | 2016 |  | IFAT | ≥1:16 | 2013-2015 | January-October 2013 August 2014-April 2015 | 35 | 25.7 | Domestic cats | 8 | [Arraes-Santos et al](http://www.sciencedirect.com/science/article/pii/S2405939016300181#!). [197] |
| Brazil | | 2016 |  | IFAT | ≥1:16 |  |  | 31 | 58 | Clinically healthy indoor cats with outdoor access | 8 | Melo et al. [198] |
| Brazil | | 2016 |  | IEISP |  | 2015 | January-March 2015 | 102 | 0 | Cats | 10 | Teixeira et al. [199] |
| Brazil | | 2017 |  | MAT | ≥1:20 | 2014-2015 |  | 372 | 9.7 | Domestic cats | 10 | Bolais et al. [200] |
| Brazil | | 2017 |  | IFAT | ≥1:16 |  |  | 595 | 64.4 | Cats sold at markets | 6 | Magalhaes et al. [201] |
| Brazil | | 2017 |  | IFAT | ≥1:64 | 2012-2013 | February 2012-April 2013 | 231 | 45.4 | Owned and stray cats | 8 | Munhoz et al. [202] |

*Abbreviations:* MAT, modified agglutination test; SFT, Sabin and Feldman Test; LAT, latex agglutination test; ELISA, enzyme-linked immunosorbent assay; IFAT, indirect fluorescent antibody test; IHA, Indirect hemagglutination assay*;* MBDT, methylene blue dye test; DT, Dye-test; GLIPS, Gaussia luciferase immunoprecipitation system; DMA, Direct microagglutination assay; DAT, direct agglutination test; IELISA, Indirect enzyme-linked immunosorbent assay; IEISP, Indirect enzyme immunoassay solid phase

**References**

1. Afonso E, Thulliez P, Pontier D, Gilot-Fromont E. Toxoplasmosis in prey species and consequences for prevalence in feral cats: not all prey species are equal. Parasitology. 2007;134 Pt.14:1963-71.

2. Arene FOI. The prevalence and public health significanceof *Toxoplasma gondii* in domestic cats in the Niger Delta. Public Health. 1984;98:333-5.

3. Al-Kappany YM, Rajendran C, Ferreira LR, Kwok OCH, Abu-Elwafa SA, Hilali M, et al. High prevalence of toxoplasmosis in cats from Egypt: Isolation of viable *Toxoplasma gondii*, tissue distribution, and isolate designation. J Parasitol. 2010;96:1115-8.

4. Kamani J, Mani AU, Kumshe HA, Yidawi JP, Egwu GO. Prevalence of *Toxoplasma gondii* antibodies in cats in Maiduguri, Northeastern Nigeria. Acta Parasitol. 2010;55:94-5.

5. Lobetti R, Lappin MR. Prevalence of *Toxoplasma gondii*, *Bartonella* species and *haemoplasma* infection in cats in South Africa. J Feline Med Surg. 2012;14:857-62.

6. Dubey JP, Darrington C, Tiao N, Ferreira LR, Choudhary S, Molla B, et al. Isolation of viable *Toxoplasma gondii* from tissues and feces of cats from Addis Ababa, Ethiopia. J Parasitol. 2013;99:56-8.

7. Tiao N, Darrington C, Molla B, Saville WJ, Tilahun G, Kwok OC, et al. An investigation into the seroprevalence of *Toxoplasma gondii*, *Bartonella* spp., feline immunodeficiency virus (FIV), and feline leukaemia virus (FeLV) in cats in Addis Ababa, Ethiopia. Epidemiol Infect. 2013;141:1029-33.

8. Hammond-Aryee K, Van Helden LS, Van Helden PDJOJoVR. The prevalence of antibodies to *Toxoplasma gondii* in sheep in the Western Cape, South Africa. Onderstepoort J Vet Res. 2015;82:01-5.

9. Ayinmode A, Oluwayelu D, Babalola E, Lawani MJBJoVM. Serologic survey of *Toxoplasma gondii* antibodies in cats (*Felis catus*) sold at Live Animal Markets in Southwestern Nigeria. BJVM. 2017;20.

10. Lopes AP, Oliveira AC, Granada S, Rodrigues FT, Papadopoulos E, Schallig H, et al. Antibodies to *Toxoplasma gondii* and *Leishmania* spp. in domestic cats from Luanda, Angola. Vet Parasitol. 2017;239:15-8.

11. Yekkour F, Aubert D, Mercier A, Murat JB, Khames M, Nguewa P, et al. First genetic characterization of *Toxoplasma gondii* in stray cats from Algeria. Vet Parasitology. 2017;239:31-6.

12. Katsube Y, Hagiwara T, Veda K, Miyakawa H, Imaizumi K, Hanaki T, et al. Studies on toxoplasmosis. Jpn J Med Sci Biol. 1967;20:413-9.

13. Werner JK, Walton BC. Prevalence of naturally occurring *Toxoplasma gondii* infections in cats from U.S. military installations in Japan. J Parasitol. 1972;58:1148-50.

14. Durfee PT, Sung HT, Ma CH, Tsai CS, Cross JH. Serologic study of toxoplasmosis in Taiwan. Southeast Asian J Trop Med Public Health. 1975;6:170-4.

15. Durfee PT, Cross JH, Rustam, Susanto. Toxoplasmosis in man and animals in South Kalimantan (Borneo), Indonesia. Am J Trop Med Hyg. 1976;25:42-7.

16. Teutsch SM, Juranek DD, Sulzer A, Dubey JP, Sikes RK. Epidemic toxoplasmosis associated with infected cats. N Engl J Med. 1979;300:695-9.

17. Ohshima S, Tsubota N, Hiraoka K. Latex agglutination microtiter test for diagnosis of toxoplasma infection in animals. [Zentralbl Bakteriol Mikrobiol Hyg A.](https://www.ncbi.nlm.nih.gov/pubmed/7197862) 1981;250:376-82.

18. Chhabra MB, Gupta SL, Gautam OP. *Toxoplasma* seroprevalence in animals in northern India. Int J Zoonoses. 1985;12:136-42.

19. Deeb BJ, Sufan MM, DiGiacomo RF. *Toxoplasma gondii* infection of cats in Beirut, Lebanon. J Trop Med Hyg. 1985;88:301-6.

20. Lappin MR, Greene CE, Prestwood AK, Dawe DL, Marks A. Prevalence of *Toxoplasma gondii* infection in cats in Georgia using enzyme-linked immunosorbent assays for IgM, IgG, and antigens. Vet Parasitol. 1989;33:225-30.

21. Lin DS, Lai SS, Bowman DD, Jacobson RH, Barr MC, Giovengo SL. Feline immunodeficiency virus, feline leukaemia virus, *Toxoplasma gondii*, and intestinal parasitic infections in Taiwanese cats. Br Vet J. 1990;146:468-75.

22. Shen L, Zhichung L, Biaucheng Z, Huayuan Y. Prevalence of *Toxoplasma gondii* infection in man and animals in Guangdong, People's Republic of China. Vet Parasitol. 1990;34:357-60.

23. Lin D-S, Fei AC-Y, Chow H-M, Mo K-M, Pong Y-MJ師. Prevalences of antibodies to *Toxoplasma gondii* in cats and humans in Taipei, Taiwan. 1998;33:95-103.

24. Maruyama S, Hiraga S, Yokoyama E, Naoi M, Tsuruoka Y, Ogura Y, et al. Seroprevalence of *Bartonella henselae* and *Toxoplasma gondii* infections among pet cats in Kanagawa and Saitama Prefectures. J Vet Med Sci. 1998;60:997-1000.

25. Nogami S, Moritomo T, Kamata H, Tamura Y, Sakai T, Nakagaki K, et al. Seroprevalence against *Toxoplasma gondii* in domiciled cats in Japan. J Vet Med Sci. 1998;60:1001-4.

26. Sohn WM, Nam HW. Western blot analysis of stray cat sera against *Toxoplasma gondii* and the diagnostic availability of monoclonal antibodies in sandwich-ELISA. Korean J Parasitol. 1999;37:249-56.

27. Kimbita EN, Xuan X, Huang X, Miyazawa T, Fukumoto S, Mishima M, et al. Serodiagnosis of *Toxoplasma gondii* infection in cats by enzyme-linked immunosorbent assay using recombinant SAG1. Vet Parasitol. 2001;102:35-44.

28. Huang X, Xuan X, Kimbita EN, Battur B, Miyazawa T, Fukumoto S, et al. Development and evaluation of an enzyme-linked immunosorbent assay with recombinant SAG2 for diagnosis of *Toxoplasma gondii* infection in cats. J Parasitol. 2002;88:804-7.

29. Maruyama S, Kabeya H, Nakao R, Tanaka S, Sakai T, Xuan X, et al. Seroprevalence of Bartonella henselae, *Toxoplasma gondii*, FIV and FeLV infections in domestic cats in Japan. Microbiol Immunol. 2003;47:147-53.

30. Sukthana Y, Kaewkungwal J, Jantanavivat C, Lekkla A, Chiabchalard R, Aumarm W. *Toxoplasma gondii* antibody in Thai cats and their owners. Southeast Asian J Trop Med Public Health. 2003;34:733-8.

31. Huang X, Xuan X, Hirata H, Yokoyama N, Xu L, Suzuki N, et al. Rapid Immunochromatographic Test Using Recombinant SAG2 for Detection of Antibodies against *Toxoplasma gondii* in Cats. J Clin Microbiol. 2004;42:351-3.

32. Salant H, Spira DTJVP. A cross-sectional survey of anti-*Toxoplasma gondii* antibodies in Jerusalem cats. Vet Parasitol. 2004;124:167-77.

33. Haddadzadeh HR, Khazraiinia P, Aslani M, Rezaeian M, Jamshidi S, Taheri M, et al. Seroprevalence of *Toxoplasma gondii* infection in stray and household cats in Tehran. Vet Parasitol. 2006;138:211-6.

34. Shahzad A, Sarwar Khan M, Ashraf K, Avais M, Pervez K, Ali Khan JJTJop. Sero-epidemiological and haematological studies on toxoplasmosis in cats, dogs and their owners in Lahore, Pakistan. J Protozool Res. 2006;16:60-73.

35. Dubey JP, Zhu XQ, Sundar N, Zhang H, Kwok OC, Su C. Genetic and biologic characterization of *Toxoplasma gondii* isolates of cats from China. Vet Parasitol. 2007;145:352-6.

36. Hooshyar H, Rostamkhani P, Talari S, Arbabi MJIJoP. *Toxoplasma gondii* infection in stray cats. Iran J Parasitol. 2007:18-22.

37. Jittapalapong S, Nimsupan B, Pinyopanuwat N, Chimnoi W, Kabeya H, Maruyama S. Seroprevalence of *Toxoplasma gondii* antibodies in stray cats and dogs in the Bangkok metropolitan area, Thailand. Vet Parasitol. 2007;145:138-41.

38. Chandrawathani P, Nurulaini R, Zanin CM, Premaalatha B, Adnan M, Jamnah O, et al. Seroprevalence of *Toxoplasma gondii* antibodies in pigs, goats, cattle, dogs and cats in peninsular Malaysia. Trop Biomed. 2008;25:257-8.

39. Karatepe B, Babür C, Karatepe M, Kiliç S, Dündar B. Prevalence of *Toxoplasma gondii* antibodies and intestinal parasites in stray cats from Nigde, Turkey. Ital J Anim Sci. 2008;7:113-8.

40. Kim HY, Kim YA, Kang S, Lee HS, Rhie HG, Ahn HJ, et al. Prevalence of *Toxoplasma gondii* in stray cats of Gyeonggi-do, Korea. Korean J Parasitol. 2008;46:199-201.

41. Özkan AT, Çelebi B, Babür C, Lucio-Forster A, Bowman DD, Lindsay DS. Investigation of anti-*Toxoplasma gondii* antibodies in cats of the Ankara region of Turkey using the Sabin-Feldman dye test and an indirect fluorescent antibody test. J Parasitol. 2008;94:817-20.

42. Yu J, Ding J, Xia Z, Lin D, Li Y, Jia J, et al. Seroepidemiology of *Toxoplasma gondii* in pet dogs and cats in Beijing, China. Acta Parasitol. 2008;53:317-9.

43. Sharif M, Daryani A, Nasrolahei M, Ziapour S. Prevalence of *Toxoplasma gondii* antibodies in stray cats in Sari, northern Iran. Trop Anim Health Prod. 2009;41:183-7.

44. Zhang H, Zhou DH, Zhou P, Lun ZR, Chen XG, Lin RQ, et al. Seroprevalence of *toxoplasma gondii* infection in stray and household cats in Guangzhou, China. Zoonoses Public Health*.* 2009;56:502-5.

45. Advincula JKdC, Iewida SYP, Salibay CC, Medica JS. Serologic detection of *Toxoplasma gondii* infection in stray and household cats and its hematologic evaluation. Sci Med 2010;20:76-82.

46. Akhtardanesh B, Ziaali N, Sharifi H, Rezaei S. Feline immunodeficiency virus, feline leukemia virus and *Toxoplasma gondii* in stray and household cats in Kerman-Iran: Seroprevalence and correlation with clinical and laboratory findings. Res Vet Sci. 2010;89:306-10.

47. Javadi S, Rezaei SA, Tajik H, Hadian M, Shokouhi F. Haematological changes of cats with *Toxoplasma gondii*-specific antibodies. Comp Clin Path. 2010;19:307-10.

48. Jittapalapong S, Inpankaew T, Pinyopanuwat N, Chimnoi W, Kengradomkij C, Wongnarkpet S, et al. Epidemiology of *toxoplasma gondii* infection of stray cats in Bangkok, Thailand. Southeast Asian J Trop Med Public Health. 2010;41:13-8.

49. Lee SE, Kim JY, Kim YA, Cho SH, Ahn HJ, Woo HM, et al. Prevalence of *Toxoplasma gondii* infection in stray and household cats in regions of Seoul, Korea. Korean J Parasitol. 2010;48:267-70.

50. Al-Ramahi HM, Hamza RH, Abdulla MAJJBU. Seroprevalence study of Toxoplasmosis in domestic animals in Mid-Euphrates region-Iraq. College of Veterinary Medicine- Babylon University-Babylon. 2010;18:1382-7.

51. Mosallanejad B, Avizeh R, Razi Jalali MH, Pourmehdi M. A study on seroprevalence and coproantigen detection of *Toxoplasma gondii* in companion cats in Ahvaz area, southwestern Iran. Iran J Vet Res. 2011;12:139-44.

52. Al-Mohammed HI. Seroprevalence of *toxoplasma gondii* infection in cats, dogs and ruminant animals in Al-Ahsa area in Saudi Arabia. Res J Med Sci. 2011;5:190-2.

53. Chong CK, Jeong W, Kim HY, An DJ, Jeoung HY, Ryu JE, et al. Development and clinical evaluation of a rapid serodiagnostic test for toxoplasmosis of cats using recombinant SAG1 antigen. Korean J Parasitol. 2011;49:207-12.

54. Hamidinejat H, Mosalanejad B, Avizeh R, Jalali MHR, Ghorbanpour M, Namavari M. Neospora caninum and *Toxoplasma gondii* antibody prevalence in Ahvaz feral cats, Iran. Jundishapur J Microbiol. 2011;4.

55. Kulasena VA, Rajapakse RPVJ, Dubey JP, Dayawansa PN, Premawansa S. Seroprevalence of *Toxoplasma gondii* in cats from Colombo, Sri Lanka. J Parasitol. 2011;97:152.

56. Lee SE, Kim NH, Chae HS, Cho SH, Nam HW, Lee WJ, et al. Prevalence of *Toxoplasma gondii* infection in feral cats in Seoul, Korea. J Parasitol. 2011;97:153-5.

57. Raeghi S, Sedeghi S, Sedeghi S. Prevalence of *Toxoplasma gondii* antibodies in cats in Urmia, northwest of Iran. J Anim Plant Sci. 2011;21:132-4.

58. Wu SM, Zhu XQ, Zhou DH, Fu BQ, Chen J, Yang JF, et al. Seroprevalence of *Toxoplasma gondii* infection in household and stray cats in Lanzhou, northwest China. Parasit Vectors. 2011;4.

59. Qian W, Wang H, Su C, Shan D, Cui X, Yang N, et al. Isolation and characterization of *Toxoplasma gondii* strains from stray cats revealed a single genotype in Beijing, China. Vet Parasitol. 2012;187:408-13.

60. Sukhumavasi W, Bellosa ML, Lucio-Forster A, Liotta JL, Lee ACY, Pornmingmas P, et al. Serological survey of *Toxoplasma gondii*, *Dirofilaria immitis*, Feline Immunodeficiency Virus (FIV) and Feline Leukemia Virus (FeLV) infections in pet cats in Bangkok and vicinities, Thailand. Vet Parasitol. 2012;188:25-30.

61. Wang Q, Jiang W, Chen YJ, Liu CY, Shi JL, Li XT. Prevalence of *Toxoplasma gondii* antibodies, circulating antigens and DNA in stray cats in Shanghai, China. Parasit Vectors. 2012;5:1756-3305.

62. Zhu CH, Cui LL, Zhang LS. Comparison of a commercial ELISA with the modified agglutination test for detection of *Toxoplasma gondii* antibodies in sera of naturally infected dogs and cats. Iran J Parasitol. 2012;7:89-95.

63. Abdou NE, Al-Batel MK, El-Azazy OM, Sami AM, Majeed QA. Enteric protozoan parasites in stray cats in Kuwait with special references to toxoplasmosis and risk factors affecting its occurrence. J Egypt Soc Parasitol. 2013;43:303-14.

64. Arunvipas P, Jittapalapong S, Inpankaew T, Pinyopanuwat N, Chimnoi W, Maruyama SJAJoAR. Seroprevalence and risk factors influenced transmission of *Toxoplasma gondii* in dogs and cats in dairy farms in Western Thailand. Afr J Agric Res. 2013;8:591-5.

65. Fuh YB, Lin CS, Liao AT, Pong YM, Tung MC, Fei CY, et al. Survey of *Toxoplasma gondii* in Taipei: Livestock meats, internal organs, cat and dog sera. TJVM. 2013;43:15-21.

66. Hong SH, Jeong YI, Kim JY, Cho SH, Lee WJ, Lee SE. Prevalence of *Toxoplasma gondii* infection in household cats in Korea and risk factors. Korean J Parasitol. 2013;51:357-61.

67. Reyes MF, Guevara VG, San Roque DGD, Flores MLS, Lastica EAJPJoV, Sciences A. Seroprevalence of *Toxoplasma gondii* antibodies in domestic short-haired cats (*Felis catus*) in a wildlife facility in Manila. Philipp J Vet Anim Sci. 2013;39.

68. Switzer AD, McMillan-Cole AC, Kasten RW, Stuckey MJ, Kass PH, Chomel BB. *Bartonella* and *Toxoplasma* infections in stray cats from Iraq. Am J Trop Med Hyg. 2013;89:1219-24.

69. Ahmad N, Qayyum M. Seroprevalence and risk factors for toxoplasmosis in large ruminants in northern Punjab, Pakistan. J Infect Dev Ctries. 2014;8:1022-8.

70. Can H, Doskaya M, Ajzenberg D, Ozdemir HG, Caner A, Iz SG, et al. Genetic characterization of *Toxoplasma gondii* isolates and toxoplasmosis seroprevalence in stray cats of Izmir, Turkey. PLoS One. 2014;9.

71. Derakhshan M, Mousavi M. Serological survey of antibodies to *Toxoplasma gondii* in cats, goats, and sheep in Kerman, Iran. Comp Clin Path. 2014;23:267-8.

72. Liu QX, Wang S, Wang LQ, Xing J, Gao WJ, Liu GF, et al. Seroprevalence of *Toxoplasma gondii* infection in dogs and cats in Zhenjiang City, Eastern China. Asian Pac J Trop Biomed. 2014;4:725-8.

73. Park HJ, Lee SE, Hong SH, Lee WJ, Seo KW, Song KH. Seroprevalence of *Toxoplasma gondii* and *Bartonella henselase* infection in stray cats of the Daejeon City, Korea. Korean J Vet Res. 2014;54:87-9.

74. Jiang W, Liu Y, Chen Y, Yang Q, Chun P, Yao K, et al. A novel dynamic flow immunochromatographic test (DFICT) using gold nanoparticles for the serological detection of *Toxoplasma gondii* infection in dogs and cats. Biosens Bioelectron. 2015;72:133-9.

75. Cai Y, Wang Z, Li J, Li N, Wei F, Liu Q. Evaluation of an indirect elisa using recombinant granule antigen Gra7 for serodiagnosis of *toxoplasma gondii* infection in cats. J Parasitol. 2015;101:37-40.

76. Oi M, Yoshikawa S, Maruyama S, Nogami S. Comparison of *Toxoplasma gondii* seroprevalence in shelter cats and dogs during 1999-2001 and 2009-2011 in Tokyo, Japan. PLoS One. 2015;10.

77. Tehrani-Sharif M, Jahan S, Alavi SM, Khodami M. Seroprevalence of *Toxoplasma gondii* antibodies of stray cats in Garmsar, Iran. J Parasit Dis. 2015;39:306-8.

78. Yang R, Ying JL, Monis P, Ryan U. Molecular characterisation of *Cryptosporidium* and *Giardia* in cats (*Felis catus*) in Western Australia. Exp Parasitol. 2015;155:13-8.

79. Pavlova EV, Kirilyuk EV, Naidenko SV. Occurrence Pattern of Influenza A Virus, *Coxiella burnetii*, *Toxoplasma gondii*, and *Trichinella* sp. in the Pallas Cat and Domestic Cat and Their Potential Prey Under Arid Climate Conditions. Arid Ecosystems. 2016;6:277-83.

80. Cong W, Meng QF, Blaga R, Villena I, Zhu XQ, Qian AD. *Toxoplasma gondii*, *Dirofilaria immitis*, feline immunodeficiency virus (FIV), and feline leukemia virus (FeLV) infections in stray and pet cats (*Felis catus*) in northwest China: co-infections and risk factors. Parasitol Res. 2016;115:217-23.

81. Erkiliç EE, Mor N, Babür C, Kırmızıgül AH, Beyhan YE. The seroprevalence of *Toxoplasma gondii* in cats from the Kars Region, Turkey. ISR J VET MED. 2016;71:31-5.

82. Kang YH, Cong W, Qin SY, Shan XF, Gao YH, Wang CF, et al. First Report of *Toxoplasma gondii*, *Dirofilaria immitis*, and *Chlamydia felis* Infection in Stray and Companion Cats in Northeastern and Eastern China. Vector Borne Zoonotic Dis. 2016;16:654-8.

83. Yang YR, Feng YJ, Lu YY, Dong H, Li TY, Jiang YB, et al. Antibody Detection, Isolation, Genotyping, and Virulence of *Toxoplasma gondii* in Captive Felids from China. Front Microbiol. 2017;8.

84. Abdelbaset AE, Alhasan H, Salman D, Karram MH, Ellah Rushdi MA, Xuenan X, et al. Evaluation of recombinant antigens in combination and single formula for diagnosis of feline toxoplasmosis. Exp Parasitol. 2017;172:1-4.

85. Boughattas S, Behnke J, Sharma A, Abu-Madi M. Seroprevalence of *Toxoplasma gondii* infection in feral cats in Qatar. BMC Vet Res. 2017;13:017-0952.

86. Hwang J, Gottdenker N, Oh DH, Lee H, Chun MS. Infections by pathogens with different transmission modes in feral cats from urban and rural areas of Korea. J Vet Sci. 2017;18:541-5.

87. Kim SE, Choi R, Kang SW, Hyun C. Prevalence of *Toxoplasma gondii* infection in household and feral cats in Korea. J Parasit Dis. 2017;41:823-5.

88. Matsuu A, Yokota SI, Ito K, Masatani T. Seroprevalence of *Toxoplasma gondii* in free-ranging and feral cats on Amami Oshima Island, Japan. J Vet Med Sci. 2017;79:1853-6.

89. Mosallanejad B, Hamidinejat H, Seifiabad Shapouri MR, Rezaei Ghaleh F. A comparison between serological and molecular tests in diagnosis of *Toxoplasma gondii* infection among stray cats in Ahvaz, southwestern Iran. Archives of Razi Institute. 2017;72:107-14.

90. Wang S, Zhou Y, Niu J, Xie Q, Xiao T, Chen Y, et al. Seroprevalence of *Toxoplasma gondii* infection in domestic cats in central China. Parasite. 2017;24.

91. Jakob‐Hoff RM, Dunsmore JD. Epidemiological aspects of toxoplasmosis in southern Western Australia. Aust Vet J. 1983;60:217-8.

92. Sumner B, Ackland ML. *Toxoplasma gondii* antibody in domestic cats in Melbourne. Aust Vet J. 1999;77:447-9.

93. Fancourt BA. Rapid decline in detections of the *Tasmanian bettong* (*Bettongia gaimardi*) following local incursion of feral cats (*Felis catus*). Aust Mammal. 2014;36:247-53.

94. Kapperud G. Survey for toxoplasmosis in wild and domestic animals from Norway and Sweden. J Wildl Dis. 1978;14:157-62.

95. Gethings PM, Stephens GL, Wills JM, Howard P, Balfour AH, Wright AI, et al. Prevalence of *chlamydia, toxoplasma, toxocara* and ringworm in farm cats in south-west England. Vet Rec. 1987;121:213-6.

96. Jackson MH, Hutchison WM, Siim JC. Prevalence of *Toxoplasma gondii* in meat animals, cats and dogs in central Scotland. Br Vet J. 1987;143:159-65.

97. Uggla A, Mattson S, Juntti N. Prevalence of antibodies to *Toxoplasma gondii* in cats, dogs and horses in Sweden. Acta Vet Scand. 1990;31:219-22.

98. Tenter AM, Vietmeyer C, Johnson AM, Janitschke K, Rommel M, Lehmacher W. ELISAs based on recombinant antigens for seroepidemiological studies on *Toxoplasma gondii* infections in cats. Parasitology. 1994;109:29-36.

99. Yamaguchi N, Macdonald DW, Passanisi WC, Harbour DA, Hopper CD. Parasite prevalence in free-ranging farm cats, Felis silvestris catus. Epidemiol Infect. 1996;116:217-23.

100. D'Amore E, Falcone E, Busani L, Tollis M. A serological survey of feline immunodeficiency virus and *Toxoplasma gondii* in stray cats. Vet Res Commun 1997;21:355-9.

101. Svobodova V, Knotek Z, Svoboda M. Prevalence of IgG and IgM antibodies specific to *Toxoplasma gondii* in cats. Vet Parasitol. 1998;80:173-6.

102. Dorny P, Speybroeck N, Verstraete S, Baeke M, De Becker A, Berkvens D, et al. Serological survey of *Toxoplasma gondii*, feline immunodeficiency virus and feline leukaemia virus in urban stray cats in Belgium. Vet Rec. 2002;151:626-9.

103. Smielewska-Loś E, Pacoń J. *Toxoplasma gondii* infection of cats in epizootiological and clinical aspects. Pol J Vet Sci. 2002;5:227-30.

104. Gauss CB, Almeria S, Ortuno A, Garcia F, Dubey JP. Seroprevalence of *Toxoplasma gondii* antibodies in domestic cats from Barcelona, Spain. J Parasitol. 2003;89:1067-8.

105. Porqueddu M, Scala A, Tilocca V. Principal endoparasitoses of domestic cats in Sardinia. Vet Res Commun. 2004;28:311-3.

106. Miró G, Montoya A, Jiménez S, Frisuelos C, Mateo M, Fuentes I. Prevalence of antibodies to *Toxoplasma gondii* and intestinal parasites in stray, farm and household cats in Spain. Vet Parasitol. 2004;126:249-55.

107. Papini R, Sbrana C, Rosa B, Saturni A, Sorrentino A, Cerretani M, et al. Serological survey of *Toxoplasma gondii* infections in stray cats from Italy. Rev Med Vet. 2006;157:193.

108. Afonso E, Thulliez P, Gilot-Fromont E. Transmission of *Toxoplasma gondii* in an urban population of domestic cats (*Felis catus*). Int J Parasitol. 2006;36:1373-82.

109. Meunier V, Jourda S, Deville M, Guillot J. Prevalence of anti-*Toxoplasma gondii* antibodies in serum and aqueous humor samples from cats with uveitis or systemic diseases in France. Vet Parasitol. 2006;138:362-5.

110. Sedlak K, Bartova E. The prevalence of *Toxoplasma gondii* IgM and IgG antibodies in dogs and cats from the Czech Republic. Vet Med. 2006;51:555-8.

111. Natale A, Frangipane di Regalbono A, Zanellato G, Cavalletto M, Danesi P, Capelli G, et al. Parasitological survey on stray cat colonies from the Veneto region. Vet Res Commun. 2007;31:241-4.

112. Sroka J, Zwoliński J, Dutkiewicz J. Seroprevalence of *Toxoplasma gondii* in farm and wild animals from the area of Lublin province. Bull Vet Inst Pulawy. 2007;51:535-40.

113. Hornok S, Edelhofer R, Joachim A, Farkas R, Berta K, Répási A, et al. Seroprevalence of *Toxoplasma gondii* and *Neospora caninum* infection of cats in Hungary. Acta Vet Hung. 2008;56:81-8.

114. Lopes AP, Cardoso L, Rodrigues M. Serological survey of *Toxoplasma gondii* infection in domestic cats from northeastern Portugal. Vet Parasitol. 2008;155:184-9.

115. Montoya A, Miró G, Mateo M, Ramírez C, Fuentes I. Molecular characterization of *Toxoplasma gondii* isolates from cats in Spain. J Parasitol. 2008;94:1044-6.

116. Macri G, Sala M, Linder AM, Pettirossi N, Scarpulla M. Comparison of indirect fluorescent antibody test and modified agglutination test for detecting *Toxoplasma gondii* immunoglobulin G antibodies in dog and cat. Parasitol Res. 2009;105:35-40.

117. Millan J, Cabezon O, Pabon M, Dubey JP, Almeria S. Seroprevalence of *Toxoplasma gondii* and *Neospora caninum* in feral cats (*Felis silvestris catus*) in Majorca, Balearic Islands, Spain. Vet Parasitol. 2009;165:323-6.

118. Afonso E, Thulliez P, Gilot-Fromont E. Local meteorological conditions, dynamics of seroconversion to *Toxoplasma gondii* in cats (*Felis catus*) and oocyst burden in a rural environment. Epidemiol Infect. 2010;138:1105-13.

119. Duarte J, Pacheco MTT, Villaverde AB, Machado RZ, Zângaro RA, Silveira Jr L. Near-infrared Raman spectroscopy to detect anti-*Toxoplasma gondii* antibody in blood sera of domestic cats: Quantitative analysis based on partial least-squares multivariate statistics. J Biomed Opt. 2010;15.

120. Juvet F, Lappin MR, Brennan S, Mooney CT. Prevalence of selected infectious agents in cats in Ireland. J Feline Med Surg. 2010;12:476-82.

121. Mancianti F, Nardoni S, Ariti G, Parlanti D, Giuliani G, Papini RA. Cross-sectional survey of *Toxoplasma gondii* infection in colony cats from urban Florence (Italy). J Feline Med Surg. 2010;12:351-4.

122. Michalski MM, Platt-Samoraj A, Mikulska-Skupien E. *Toxoplasma gondii* antibodies in domestic cats in Olsztyn urban area, Poland. Wiad Parazytol. 2010;56:277-9.

123. Bennett AD, Gunn-Moore DA, Brewer M, Lappin MR. Prevalence of Bartonella species, haemoplasmas and *Toxoplasma gondii* in cats in Scotland. J Feline Med Surg. 2011;13:553-7.

124. Darabus G, Hotea I, Oprescit I, Morariu S, Brudiu I, Olariu RT. Toxoplasmosis seroprevalence in cats and sheep from Western Romania. Rev Med Vet. 2011;162:316-20.

125. Györke A, Opsteegh M, Mircean V, Iovu A, Cozma V. *Toxoplasma gondii* in Romanian household cats: Evaluation of serological tests, epidemiology and risk factors. Prev Vet Med. 2011;102:321-8.

126. Jokelainen P, Simola O, Rantanen E, Nareaho A, Lohi H, Sukura A. Feline toxoplasmosis in Finland: cross-sectional epidemiological study and case series study. J Vet Diagn Invest. 2012;24:1115-24.

127. Opsteegh M, Haveman R, Swart AN, Mensink-Beerepoot ME, Hofhuis A, Langelaar MF, et al. Seroprevalence and risk factors for *Toxoplasma gondii* infection in domestic cats in The Netherlands. Prev Vet Med. 2012;104:317-26.

128. Spada E, Proverbio D, della Pepa A, Perego R, Baggiani L, DeGiorgi GB, et al. Seroprevalence of feline immunodeficiency virus, feline leukaemia virus and *Toxoplasma gondii* in stray cat colonies in northern Italy and correlation with clinical and laboratory data. J Feline Med Surg. 2012;14:369-77.

129. Waap H, Cardoso R, Leitão A, Nunes T, Vilares A, Gargaté MJ, et al. *In vitro* isolation and seroprevalence of *Toxoplasma gondii* in stray cats and pigeons in Lisbon, Portugal. Vet Parasitol. 2012;187:542-7.

130. Afonso E, Germain E, Poulle ML, Ruette S, Devillard S, Say L, et al. Environmental determinants of spatial and temporal variations in the transmission of *Toxoplasma gondii* in its definitive hosts. Int J Parasitol Parasites Wildl. 2013;2:278-85.

131. Deksne G, Petruseviča A, Kirjušina M. Seroprevalence and factors associated with *Toxoplasma gondii* infection in domestic cats from urban areas in Latvia. J Parasitol*.* 2013;99:48-50.

132. Spada E, Proverbio D, Della Pepa A, Domenichini G, Bagnagatti De Giorgi G, Traldi G, et al. Prevalence of faecal-borne parasites in colony stray cats in northern Italy. J Feline Med Surg. 2013;15:672-7.

133. Esteves F, Aguiar D, Rosado J, Costa ML, de Sousa B, Antunes F, et al. *Toxoplasma gondii* prevalence in cats from Lisbon and in pigs from centre and south of Portugal. Vet Parasitol. 2014;200:8-12.

134. Silaghi C, Knaus M, Rapti D, Kusi I, Shukullari E, Hamel D, et al. Survey of *Toxoplasma gondii* and *Neospora caninum*, *haemotropic mycoplasmas* and other arthropod-borne pathogens in cats from Albania. Parasit Vectors. 2014;7:1756-3305.

135. Must K, Lassen B, Jokelainen P. Seroprevalence of and Risk Factors for *Toxoplasma gondii* Infection in Cats in Estonia. Vector Borne Zoonotic Dis. 2015;15:597-601.

136. Sævik BK, Krontveit RI, Eggen KP, Malmberg N, Thoresen SI, Prestrud KW. *Toxoplasma gondii* seroprevalence in pet cats in Norway and risk factors for seropositivity. J Feline Med Surg*.* 2015;17:1049-56.

137. Spada E, Canzi I, Baggiani L, Perego R, Vitale F, Migliazzo A, et al. Prevalence of *Leishmania infantum* and co-infections in stray cats in northern Italy. Comp Immunol Microbiol Infect Dis. 2016;45:53-8.

138. Veronesi F, Santoro A, Milardi GL, Diaferia M, Morganti G, Ranucci D, et al. Detection of *Toxoplasma gondii* in faeces of privately owned cats using two PCR assays targeting the B1 gene and the 529-bp repetitive element. Parasitol Res. 2017;116:1063-9.

139. Tizard IR, Harmeson J, Lai CH. The prevalence of serum antibodies to *Toxoplasma gondii* in Ontario mammals. Can J Comp Med. 1978;42:177-83.

140. Ladiges WC, DiGiacomo RF, Yamaguchi RA. Prevalence of *Toxoplasma gondii* antibodies and oocysts in pound-source cats. J Am Vet Med Assoc*.* 1982;180:1334-5.

141. Rodgers SJ, Baldwin CA. A serologic survey of Oklahoma cats for antibodies to feline immunodeficiency virus, coronavirus, and *Toxoplasma gondii* and for antigen to feline leukemia virus. J Vet Diagn Invest. 1990;2:180-3.

142. Smith KE, Zimmerman JJ, Patton S, Beran GW, Hill HT. The epidemiology of toxoplasmosis on Iowa swine farms with an emphasis on the roles of free-living mammals. Vet Parasitol. 1992;42:199-211.

143. Frenkel JK, Hassanein KM, Hassanein RS, Brown E, Thulliez P, Quintero-Nunez R. Transmission of *Toxoplasma gondii* in Panama City, Panama: a five-year prospective cohort study of children, cats, rodents, birds, and soil. Am J Trop Med Hyg. 1995;53:458-68.

144. Hill SL, Cheney JM, Taton-Allen GF, Reif JS, Bruns C, Lappin MR. Prevalence of enteric zoonotic organisms in cats. J Am Vet Med Assoc. 2000;216:687-92.

145. DeFeo ML, Dubey JP, Mather TN, Rhodes RC, 3rd. Epidemiologic investigation of seroprevalence of antibodies to *Toxoplasma gondii* in cats and rodents. Am J Vet Res. 2002;63:1714-7.

146. Dubey JP, Saville WJA, Stanek JF, Reed SM. Prevalence of *Toxoplasma gondii* antibodies in domestic cats from rural Ohio. J Parasitol. 2002;88:802-3.

147. Rossano MG, Murphy AJ, Vrable RA, Vanzo NE, Lewis SK, Sheline KD, et al. Cross-sectional study of serum antibodies against Sarcocystis neurona in cats tested for antibodies against *Toxoplasma gondii*. J Am Vet Med Assoc. 2002;221:511-4.

148. Nutter FB, Dubey JP, Levine JF, Breitschwerdt EB, Ford RB, Stoskopf MK. Seroprevalences of antibodies against *Bartonella henselae* and *Toxoplasma gondii* and fecal shedding of *Cryptosporidium* spp, *Giardia* spp, and *Toxocara cati* in feral and pet domestic cats. J Am Vet Med Assoc. 2004;225:1394-8.

149. Vollaire MR, Radecki SV, Lappin MRJAjovr. Seroprevalence of *Toxoplasma gondii* antibodies in clinically ill cats in the United States. Am J Vet Res. 2005;66:874-7.

150. Lickey AL, Kennedy M, Patton S, Ramsay EC. Serologic survey of domestic felids in the Peten region of Guatemala. J Zoo Wildl Med. 2005;36:121-3.

151. Asthana SP, Macpherson CN, Weiss SH, Stephens R, Denny TN, Sharma RN, et al. Seroprevalence of *Toxoplasma gondii* in pregnant women and cats in Grenada, West Indies. J Parasitol. 2006;92:644-5.

152. García-Márquez LJ, Gutiérrez-Díaz MA, Correa D, Luna-Pastén H, Palma JMJJoP. Prevalence of *Toxoplasma gondii* antibodies and the relation to risk factors in cats of Colima, Mexico. [J Parasitol.](https://www.ncbi.nlm.nih.gov/pubmed/18314706) 2007;93:1527-8.

153. Alvarado-Esquivel C, Liesenfeld O, Herrera-Flores RG, Ramírez-Sánchezf BE, González-Herrera A, Martínez-García SA, et al. Seroprevalence of *Toxoplasma gondii* antibodies in cats from Durango City, Mexico. J Parasitol. 2007;93:1214-6.

154. Dabritz HA, Gardner IA, Miller MA, Lappin MR, Atwill ER, Packham AE, et al. Evaluation of two *Toxoplasma gondii* serologic tests used in a serosurvey of domestic cats in California. J Parasitol. 2007;93:806-16.

155. Danner RM, Goltz DM, Hess SC, Banko PC. Evidence of feline immunodeficiency virus, feline leukemia virus, and *Toxoplasma gondii* in feral cats on Mauna Kea, Hawaii. J Wildl Dis. 2007;43:315-8.

156. Moura L, Kelly P, Krecek RC, Dubey JP. Seroprevalence of *Toxoplasma gondii* in cats from St. Kitts, West Indies. J Parasitol. 2007;93:952-3.

157. de Camps S, Dubey JP, Saville WJ. Seroepidemiology of *Toxoplasma gondii* in zoo animals in selected zoos in the midwestern United States. J Parasitol. 2008;94:648-53.

158. Besné-Mérida A, Figueroa-Castillo JA, Martínez-Maya JJ, Luna-Pastén H, Calderón-Segura E, Correa D. Prevalence of antibodies against *Toxoplasma gondii* in domestic cats from Mexico City. Vet Parasitol. 2008;157:310-3.

159. Dubey JP, Lappin MR, Kwok OC, Mofya S, Chikweto A, Baffa A, et al. Seroprevalence of *Toxoplasma gondii* and concurrent *Bartonella* spp., feline immunodeficiency virus, and feline leukemia virus infections in cats from Grenada, West Indies. J Parasitol. 2009;95:1129-33.

160. Dubey JP, Moura L, Majumdar D, Sundar N, Velmurugan GV, Kwok OC, et al. Isolation and characterization of viable *Toxoplasma gondii* isolates revealed possible high frequency of mixed infection in feral cats ( *Felis domesticus*) from St Kitts, West Indies. Parasitology. 2009;136:589-94.

161. Dubey JP, Bhatia CR, Lappin MR, Ferreira LR, Thorn A, Kwok OC. Seroprevalence of *Toxoplasma gondii* and *Bartonella* spp. antibodies in cats from Pennsylvania. J Parasitol. 2009;95:578-80.

162. Dubey JP, Velmurugan GV, Alvarado-Esquivel C, Alvarado-Esquivel D, Rodriguez-Pena S, Martinez-Garcia S, et al. Isolation of *Toxoplasma gondii* from animals in Durango, Mexico. J Parasitol. 2009;95:319-22.

163. Powell CC, McInnis CL, Fontenelle JP, Lappin MR. Bartonella species, feline herpesvirus-1, and *Toxoplasma gondii* PCR assay results from blood and aqueous humor samples from 104 cats with naturally occurring endogenous uveitis. J Feline Med Surg. 2010;12:923-8.

164. Hsu V, Grant DC, Zajac AM, Witonsky SG, Lindsay DS. Prevalence of IgG antibodies to *Encephalitozoon cuniculi* and *Toxoplasma gondii* in cats with and without chronic kidney disease from Virginia. Vet Parasitol. 2011;176:23-6.

165. Castillo-Morales VJ, Acosta Viana KY, Guzmán-Marín EDS, Jiménez-Coello M, Segura-Correa JC, Aguilar-Caballero AJ, et al. Prevalence and risk factors of *toxoplasma gondii* infection in domestic cats from the tropics of mexico using serological and molecular tests. Interdiscip Perspect Infect Dis. 2012; 2012:529108.

166. Jimenez-Coello M, Acosta-Viana KY, Guzman-Marin E, Gutierrez-Ruiz EJ, Rodriguez-Vivas RI, Bolio-González ME, et al. The occurrence of *Toxoplasma gondii* antibodies in backyard pigs and cats from an endemic tropical area of mexico. Tropical and Subtropical Agroecosystems. 2013;16:89-92.

167. Ballash GA, Dubey JP, Kwok OC, Shoben AB, Robison TL, Kraft TJ, et al. Seroprevalence of *Toxoplasma gondii* in White-Tailed Deer (*Odocoileus virginianus*) and Free-Roaming Cats (*Felis catus*) Across a Suburban to Urban Gradient in Northeastern Ohio. Ecohealth. 2015;12:359-67.

168. Rico-Torres CP, Del Viento-Camacho A, Caballero-Ortega H, Besne-Merida A, Luna-Pasten H, Correa D, et al. First isolation of *Toxoplasma gondii* from cats of Colima, Mexico: tissue distribution and genetic characterization. Vet Parasitol. 2015;209:125-8.

169. Rengifo-Herrera C, Pile E, García A, Pérez A, Pérez D, Nguyen FK, et al. Seroprevalence of *Toxoplasma gondii* in domestic pets from metropolitan regions of Panama. Parasite. 2017;24.

170. Fernández F, Ouviña G, Clot E, Fernandes Guido R, Codoni C. Prevalence of *Toxoplasma gondii* antibodies in cats in the western part of Great Buenos Aires, Argentina, 1993. Vet Parasitol. 1995;59:75-9.

171. Lucas SRR, Hagiwara MK, De Loureiro VS, Ikesaki JYH, Birgel EH*. Toxoplasma gondii* infection in Brazilian domestic outpatient cats. Rev Inst Med Trop Sao Paulo. 1999;41:221-4.

172. Silva JC, Gennari SM, Ragozo AM, Amajones VR, Magnabosco C, Yai LE, et al. Prevalence of *Toxoplasma gondii* antibodies in sera of domestic cats from Guarulhos and Sao Paulo, Brazil. J Parasitol. 2002;88:419-20.

173. Dubey JP, Navarro IT, Sreekumar C, Dahl E, Freire RL, Kawabata HH, et al. *Toxoplasma gondii* infections in cats from Parana, Brazil: seroprevalence, tissue distribution, and biologic and genetic characterization of isolates. J Parasitol. 2004;90:721-6.

174. Meireles LR, Galisteo AJ, Jr., Pompeu E, Andrade HF, Jr. *Toxoplasma gondii* spreading in an urban area evaluated by seroprevalence in free-living cats and dogs. Trop Med Int Health. 2004;9:876-81.

175. Cavalcante GT, Aguiar DM, Chiebao D, Dubey JP, Ruiz VLA, Dias RA, et al. Seroprevalence of *Toxoplasma gondii* antibodies in cats and pigs from Rural Western Amazon, Brazil. J Parasitol. 2006;92:863-4.

176. Dubey JP, Su C, Cortes JA, Sundar N, Gomez-Marin JE, Polo LJ, et al. Prevalence of *Toxoplasma gondii* in cats from Colombia, South America and genetic characterization of T. gondii isolates. Vet Parasitol. 2006;141:42-7.

177. Pena HF, Soares RM, Amaku M, Dubey JP, Gennari SM. *Toxoplasma gondii* infection in cats from Sao Paulo state, Brazil: seroprevalence, oocyst shedding, isolation in mice, and biologic and molecular characterization. Res Vet Sci. 2006;81:58-67.

178. Bresciani KDS, Gennari SM, Serrano ACM, Rodrigues AAR, Ueno T, Franco LG, et al. Antibodies to *Neospora caninum* and *Toxoplasma gondii* in domestic cats from Brazil. Parasitol Res. 2007;100:281-5.

179. Mendes-de-Almeida F, Labarthe N, Guerrero J, Faria MC, Branco AS, Pereira CD, et al. Follow-up of the health conditions of an urban colony of free-roaming cats (Felis catus Linnaeus, 1758) in the city of Rio de Janeiro, Brazil. Vet Parasitol. 2007;147:9-15.

180. Rosa LD, de Moura AB, Trevisani N, Medeiros AP, Sartor AA, da Souza AP, et al. *Toxoplasma gondii* antibodies on domiciled cats from Lages municipality, Santa Catarina State, Brazil. Rev Bras Parasitol Vet. 2010;19:268-9.

181. López C, Daprato B, Zampolini S, Mazzeo C, Cardillo N, Sommerfelt IJRI-LP. Risk factors and prevalence of IgG antibodies to *Toxoplasma gondii* in domestic cats: La Matanza, Buenos Aires, Argentina. Parasitol. 2011;70:29-34.

182. Coelho WMD, Do Amarante AFT, De Carvalho Apolinário J, Coelho NMD, De Lima VMF, Perri SHV, et al. Seroepidemiology of *Toxoplasma gondii*, *Neospora caninum*, and *Leishmania* spp. infections and risk factors for cats from Brazil. Parasitol Res. 2011;109:1009-13.

183. Cruz Mde A, Ullmann LS, Montano PY, Hoffmann JL, Langoni H, Biondo AW. Seroprevalence of *Toxoplasma gondii* infection in cats from Curitiba, Parana, Brazil. Rev Bras Parasitol Vet. 2011;20:256-8.

184. Braga MSCO, André MR, Jusi MMG, Freschi CR, Teixeira MCA, Machado RZ. Occurrence of anti-*Toxoplasma gondii* and anti-*Neospora caninum* antibodies in cats with outdoor access in São Luís, Maranhão, Brazil. Rev Bras Parasitol Vet. 2012;21:107-11.

185. Costa DG, Marvulo MF, Silva JS, Santana SC, Magalhaes FJ, Filho CD, et al. Seroprevalence of *Toxoplasma gondii* in domestic and wild animals from the Fernando de Noronha, Brazil. J Parasitol. 2012;98:679-80.

186. Sobrinho LS, Rossi CN, Vides JP, Braga ET, Gomes AA, de Lima VM, et al. Coinfection of *Leishmania chagasi* with *Toxoplasma gondii*, Feline Immunodeficiency Virus (FIV) and Feline Leukemia Virus (FeLV) in cats from an endemic area of zoonotic visceral leishmaniasis. Vet Parasitol. 2012;187:302-6.

187. Cardia DFF, Camossi LG, Neto LDS, Langoni H, Bresciani KDS. Prevalence of *Toxoplasma gondii* and *Leishmania* spp. infection in cats from Brazil. Vet Parasitol. 2013;197:634-7.

188. Fournier GFdSR, Lopes MG, Marcili A, Ramirez DG, Acosta ICL, Ferreira JIGdS, et al. *Toxoplasma gondii* in domestic and wild animals from forest fragments of the municipality of Natal, northeastern Brazil. Rev Bras Parasitol Vet. 2014;23:501-8.

189. Bastos BF, Brener B, Gershony L, Willi L, Labarthe N, Pereira C, et al. Seroprevalence of *Toxoplasma gondii* (Nicole & Manceaux, 1909) and retroviral status of client-owned pet cats (Felis catus, Linnaeus, 1758) in Rio de Janeiro, Brazil. Rev Inst Med Trop Sao Paulo. 2014;56:201-3.

190. Cerro L, Rubio A, Pinedo R, Mendes-de-Almeida F, Brener B, Labarthe N. Seroprevalence of *Toxoplasma gondii* in cats (*Felis catus,* Linnaeus 1758) living in Lima, Peru. Rev Bras Parasitol Vet. 2014;23:90-3.

191. Feitosa TF, Vilela VLR, Dantas ES, Souto DVO, Pena HFJ, Athayde ACR, et al. *Toxoplasma gondii* and *Neospora caninum* in domestic cats from the Brazilian semi-arid: Seroprevalence and risk factors. Arq Bras Med Vet Zootec. 2014;66:1060-6.

192. Sousa KCMd, Herrera HM, Domingos IH, Campos JBV, Santos IMCd, Neves HH, et al. Serological detection of *Toxoplasma gondii, Leishmania infantum* and *Neospora caninum* in cats from an area endemic for leishmaniasis in Brazil. Rev Bras Parasitol Vet. 2014;23:449-55.

193. Barros RS, Menezes RC, Pereira SA, Figueiredo FB, Oliveira RVCd, Nicolau JL, et al. Feline sporotrichosis: coinfection with *Toxoplasma gondii*, feline immunodeficiency virus and feline leukemia virus in cats from an endemic area in Brazil. Acta Sci Vet. 2015; 43: 1316.

194. Furtado MM, Gennari SM, Ikuta CY, De Almeida Jácomo AT, De Morais ZM, De Jesus Pena HF, et al. Serosurvey of Smooth *Brucella, Leptospira spp.* and *Toxoplasma gondii* in Free-Ranging Jaguars (*Panthera onca*) and Domestic Animals from Brazil. PLoS One. 2015;10.

195. Toro IT, Uribe A, Brenet KCA, Contreras AAV, Wiethuchter CFJRdMV. Seroprevalencia de *Toxoplasma gondii* en gatos (*Felis catus*, Linnaeus 1758) residentes en San Carlos, Chile. Rev Med Vet. 2015; 29:23-31.

196. Figueiredo de Souza S, dos Santos Medeiros L, de Souza Belfort A, Lopes Cordeiro AL, Federle M, Pereira de Souza A, et al. *Toxoplasma gondii* antibodies in domiciled cats from rio branco Municipality, Acre State, Brazil. Epidemiologia veterinária 2015;36.

197. Arraes-Santos AI, Araújo AC, Guimarães MF, Santos JR, Pena HFJ, Gennari SM, et al. Seroprevalence of anti-*Toxoplasma gondii* and anti-*Neospora caninum* antibodies in domestic mammals from two distinct regions in the semi-arid region of Northeastern Brazil. Vet Parasitol Reg Stud Reports. 2016;5:14-8.

198. Melo RPB, Almeida JC, Lima DCV, Pedrosa CM, Magalhaes FJR, Alcantara AM, et al. Atypical *Toxoplasma gondii* genotype in feral cats from the Fernando de Noronha Island, northeastern Brazil. Vet Parasitol. 2016;224:92-5.

199. Teixeira JV, de Oliveira JLS, de Almeida DMPF, de Sousa Gonçalves L, de Oliveira FLLJRBdHeSA. Seroprevalence of feline toxoplasmosis in Teresina, Piauí, Brazil. Revista Brasileria de Higiene Sanidade Animal. 2016;10:549-55.

200. Bolais PF, Vignoles P, Pereira PF, Keim R, Aroussi A, Ismail K, et al. *Toxoplasma gondii* survey in cats from two environments of the city of Rio de Janeiro, Brazil by Modified Agglutination Test on sera and filter-paper. Parasit Vectors. 2017;10.

201. Magalhães FJR, Ribeiro-Andrade M, Souza FM, Lima Filho CDF, Biondo AW, Vidotto O, et al. Seroprevalence and spatial distribution of *Toxoplasma gondii* infection in cats, dogs, pigs and equines of the Fernando de Noronha Island, Brazil. Parasitol Int. 2017;66:43-6.

202. Munhoz AD, Hage SB, Cruz RDS, Calazans APF, Silva FL, Albuquerque GR, et al. Toxoplasmosis in cats in northeastern Brazil: Frequency, associated factors and coinfection with *Neospora caninum*, feline immunodeficiency virus and feline leukemia virus. Vet Parasitol Reg Stud Reports. 2017;8:35-8.
