# Supplementary material for: The global serological prevalence of Toxoplasma gondii in felids during the last five decades (1967–2017): a systematic review and meta-analysis
Source: Parasit Vectors. 2020 Feb 17;13:82. doi: 10.1186/s13071-020-3954-1 (PMC7026947; doi:10.1186/s13071-020-3954-1)
Supplement: Supplementary file 3 — Additional file 3: Table S3. List of countries without data on Toxoplasma gondii infection in domestic cats. [file 13071_2020_3954_MOESM3_ESM.doc]

**Additional file 3: Table S3.** List of countries without data on *Toxoplasma gondii* infection in domestic cats

| **Continent** | **Country** |
| --- | --- |
| **Africa** | Angola, Benin, Botswana, Burkina Faso, Burundi, Cameroon, Cape Verde, Central African Republic, Ceuta, Chad, Comoros, Democratic Republic of the Congo, Djibouti, Equatorial Guinea, Eritrea, Eswatini, Gabon, Ghana, Guinea, Guinea-Bissau, Ivory Coast, Kenya, Lesotho, Liberia, Libya, Madagascar, Madeira, Malawi, Mali, Mauritania, Mauritius, Mayotte, Melilla, Morocco, Mozambique, Namibia, Niger, Republic of the Congo, Réunion, Rwanda, Saint Helena, Ascension and Tristan da Cunha, São Tomé and Príncipe, Senegal, Seychelles, Sierra Leone, Somalia, South Sudan, Sudan, Tanzania, The Gambia, Togo, Tunisia, Uganda, Western Sahara, Zambia, Zimbabwe |
| **Asia** | Afghanistan, Armenia, Azerbaijan, Bahrain, Bangladesh, Bhutan, Brunei, Cambodia, Jordan, Kazakhstan, Kyrgyzstan, Laos, Maldives, Mongolia, Myanmar, Nepal, North Korea, Oman, Pakistan, Palestine, Papua New Guinea, Singapore, South Korea, Syria,Tajikistan, Turkmenistan, United Arab Emirates, Uzbekistan, Vietnam, Yemen |
| **Europe** | Andorra, Armenia, Austria, Azerbaijan, Belarus, Bosnia and Herzegovina, Bulgaria Croatia, Cyprus, Denmark, Georgia, Greece, Iceland, Kazakhstan, Liechtenstein, Lithuania, Luxembourg, Malta, Moldova, Monaco, Montenegro, North Macedonia San Marino, Serbiam, Slovakia, Slovenia, Switzerland, Ukraine, Vatican City |
| **North America** | Antigua and Barbuda, The Bahamas, Barbados, Belize, Costa Rica, Cuba, Dominica, Dominican Republic, El Salvador, Greenland, Haiti, Honduras, Jamaica, Nicaragua, Puerto Rico, San Andrés and Providencia, Saint Kitts and Nevis, Saint Lucia, Saint Vincent and the Grenadines, Trinidad and Tobago |
| **South America** | Bolivia, Bouvet Island, Ecuador, Guyana, Paraguay, Suriname, Uruguay,Venezuela |
