# Supplementary material for: The global serological prevalence of Toxoplasma gondii in felids during the last five decades (1967–2017): a systematic review and meta-analysis
Source: Parasit Vectors. 2020 Feb 17;13:82. doi: 10.1186/s13071-020-3954-1 (PMC7026947; doi:10.1186/s13071-020-3954-1)
Supplement: Supplementary file 4 — Additional file 4: Table S4. List of countries without data on Toxoplasma gondii infection in wild felids. [file 13071_2020_3954_MOESM4_ESM.doc]

**Additional file 4: Table S4.** List of countries without data on *Toxoplasma gondii* infection in wild felids

| **Continent** | **Country** |
| --- | --- |
| **Antarctica** | All country of this continent. |
| **Africa** | Algeria, Egypt, Libya, Morocco, Sudan, Tunisia,Western Sahara, Burundi, Comoros, Djibouti, Eritrea, Ethiopia, Kenya, Madagascar, Malawi, Mauritius, Mozambique, Rwanda, Seychelles, Somalia, South Sudan, Tanzania, Uganda, Zambia, Zimbabwe, Angola, Cameroon, Central African Republic, Chad, Republic of the Congo, Democratic Republic of the Congo, Equatorial, Guinea, Gabon, São Tomé and Príncipe, Botswana, Eswatini, Lesotho, Namibia, Benin, Burkina Faso, Cape Verde, The Gambia, Ghana, Guinea, Guinea-Bissau, Ivory Coast, Liberia, Mali, Mauritania, Niger, Nigeria, Saint Helena, Senegal, Sierra Leone, Togo |
| **Asia** | Afghanistan, Armenia, Azerbaijan, Bahrain, Bangladesh, Bhutan, Brunei, Cambodia, China, Cyprus, East Timor, Egypt, Georgia, India, Indonesia, Iran, Iraq, Israel, Japan, Jordan, Kazakhstan, Kuwait, Kyrgyzstan, Laos, Lebanon, Malaysia, Maldives, Mongolia, Myanmar, Nepal, North Korea, Oman, Pakistan, Palestine, Papua New Guinea, Philippines, Russia, Saudi Arabia, Singapore, South Korea, Sri Lanka, Syria, Taiwan , Tajikistan, Turkey, Turkmenistan, Uzbekistan, Vietnam, Yemen |
| **Europe** | Albania, Andorra, Armenia,  Austria, Azerbaijan, Belarus, Belgium, Bosnia and Herzegovina, Bulgaria, Croatia, Cyprus , Czech Republic, Denmark, Estonia, Finland, Georgia , Germany, Greece, Hungary, Iceland, Ireland, Italy, Kazakhstan, Latvia, Liechtenstein, Lithuania, Luxembourg, Malta, Moldova, Monaco, Montenegro, Netherlands , North Macedonia, Norway, Poland, Portugal, Romania, Russia, San Marino, Serbia, Slovakia,Slovenia, Spain, Sweden, Switzerland, Turkey, Ukraine, United Kingdom, Vatican City |
| **North America** | Antigua and Barbuda, Barbados, Belize, Costa Rica, Cuba, Dominica, Dominican Republic, El Salvador, Greenland, Grenada, Guadeloupe, Guatemala, Haiti, Honduras, Jamaica, Martinique, Mexico, Montserrat, Nicaragua, Nueva Esparta, Panama, Puerto Rico, Saint Lucia, Saint Vincent and the Grenadines, The Bahamas, Trinidad and Tobago |
| **South America** | Bolivia , Chile, Colombia, Ecuador, Guyana, Paraguay, Suriname, Uruguay, Venezuela |
| **Australia** | All country of this continent. |
